# Exploring the nexus of urban form, transport, environment and health in large-scale urban studies: a state-of-the-art scoping review

**DOI:** 10.1016/j.envres.2024.119324

**Published:** 2024-06-04

**Authors:** Georgia M.C. Dyer, Sasha Khomenko, Deepti Adlakha, Susan Anenberg, Martin Behnisch, Geoff Boeing, Manuel Esperon-Rodriguez, Antonio Gasparrini, Haneen Khreis, Michelle C. Kondo, Pierre Masselot, Robert I. McDonald, Federica Montana, Rich Mitchell, Natalie Mueller, M. Omar Nawaz, Enrico Pisoni, Rafael Prieto-Curiel, Nazanin Rezaei, Hannes Taubenböck, Cathryn Tonne, Daniel Velázquez-Cortés, Mark Nieuwenhuijsen

**Affiliations:** a Barcelona Institute for Global Health (ISGlobal), Doctor Aiguader 88, 08003, Barcelona, Spain; b Universitat Pompeu Fabra (UPF), Doctor Aiguader 88, 08003, Barcelona, Spain; c CIBER Epidemiología y Salud Pública (CIBERESP), Melchor Fern’andez Almagro, 3-5, 28029, Madrid, Spain; d Delft University of Technology, Mekelweg 5, 2628, Delft, Netherlands; e Environmental and Occupational Health Department, George Washington University, Milken Institute School of Public Health, 20052, New Hampshire Avenue, Washington, District of Colombia, United States; f Leibniz Institute of Ecological Urban and Regional Development, Weberpl 1, 01217, Dresden, Germany; g University of Southern California, 90007, Los Angeles, United States; h Hawkesbury Institute for the Environment, Western Sydney University, Locked Bag 1797, Penrith, NSW, 2751, Australia; i School of Science, Western Sydney University, Locked Bag 1797, Penrith, NSW, 2751, Australia; j Environment & Health Modelling (EHM) Lab, Department of Public Health Environments and Society, London School of Hygiene & Tropical Medicine, 15-17 Tavistock Place, WC1E 7HT, London, United Kingdom; k MRC Epidemiology Unit, Cambridge University, CB2 0AH, Cambridge, United Kingdom; l USDA-Forest Service, Northern Research Station, 100 North 20th Street, Ste 205, 19103, Philadelphia, PA, United States; m The Nature Conservancy, 4245 North Fairfax Drive Arlington, 22203, Virginia, United States; n Institute of Health and Wellbeing, University of Glasgow, 90 Byres Road, Glasgow, G20 0TY, United Kingdom; o European Commission, Joint Research Centre (JRC), 2749, Ispra, Italy; p Complexity Science Hub Vienna, Josefst¨adter Straße 39, 1080, Vienna, Austria; q University of California Santa Cruz, 1156 High Street, 95064, California, United States; r German Aerospace Centre (DLR), Earth Observation Center (EOC), 82234, Oberpfaffenhofen, Germany; s Institute for Geography and Geology, Julius-Maximilians-Universit¨at Würzburg, 97074, Würzburg, Germany

## Abstract

**Background:**

As the world becomes increasingly urbanised, there is recognition that public and planetary health relies upon a ubiquitous transition to sustainable cities. Disentanglement of the complex pathways of urban design, environmental exposures, and health, and the magnitude of these associations, remains a challenge. A state-of-the-art account of large-scale urban health studies is required to shape future research priorities and equity- and evidence-informed policies.

**Objectives:**

The purpose of this review was to synthesise evidence from large-scale urban studies focused on the interaction between urban form, transport, environmental exposures, and health. This review sought to determine common methodologies applied, limitations, and future opportunities for improved research practice.

**Methods:**

Based on a literature search, 2958 articles were reviewed that covered three themes of: urban form; urban environmental health; and urban indicators. Studies were prioritised for inclusion that analysed at least 90 cities to ensure broad geographic representation and generalisability. Of the initially identified studies, following expert consultation and exclusion criteria, 66 were included.

**Results:**

The complexity of the urban ecosystem on health was evidenced from the context dependent effects of urban form variables on environmental exposures and health. Compact city designs were generally advantageous for reducing harmful environmental exposure and promoting health, with some exceptions. Methodological heterogeneity was indicative of key urban research challenges; notable limitations included exposure and health data at varied spatial scales and resolutions, limited availability of local-level sociodemographic data, and lack of consensus on robust methodologies that encompass best research practice.

**Conclusion:**

Future urban environmental health research for evidence-informed urban planning and policies requires a multi-faceted approach. Advances in geospatial and AI-driven techniques and urban indicators offer promising developments; however, there remains a wider call for increased data availability at local-levels, transparent and robust methodologies of large-scale urban studies, and greater exploration of urban health vulnerabilities and inequities.

## 1 Introduction

Currently, almost 60% of the global population (∼4.8 billion people) live in the urban environment and by 2050 nearly seven out of ten people will inhabit cities^1,2^. There are a host of reasons attributed to the rising trend of migration and urbanisation; mainly, cities provide rich opportunities for education, employment, wealth, and innovation^3,4^. Yet cities can also be a concentrated source of environmental exposure stressors (e.g., air pollution, noise, and heat)^5,6,7^, perpetuate unhealthy lifestyles^8^, and exacerbate health inequities^9^. Concurrent with rapid urbanisation, climate change poses an additional threat to urban health and sustainability challenges^10,11^. Cities account for 75% of the world’s energy-related greenhouse gas emissions^12^ and can be a major contributor to biodiversity loss^13^. Although viewed as the principal drivers of climate change, cities also offer a large part of the solution^14,15^. In Europe, initiatives that aim to reduce greenhouse gas emissions and achieve carbon neutrality include the EU’s Green Deal^16^ and the Paris Climate Agreement^17^. These initiatives recognise the pivotal role of sustainable and liveable cities for achieving these objectives, which in turn will protect public and planetary health.

**Figure 1:**
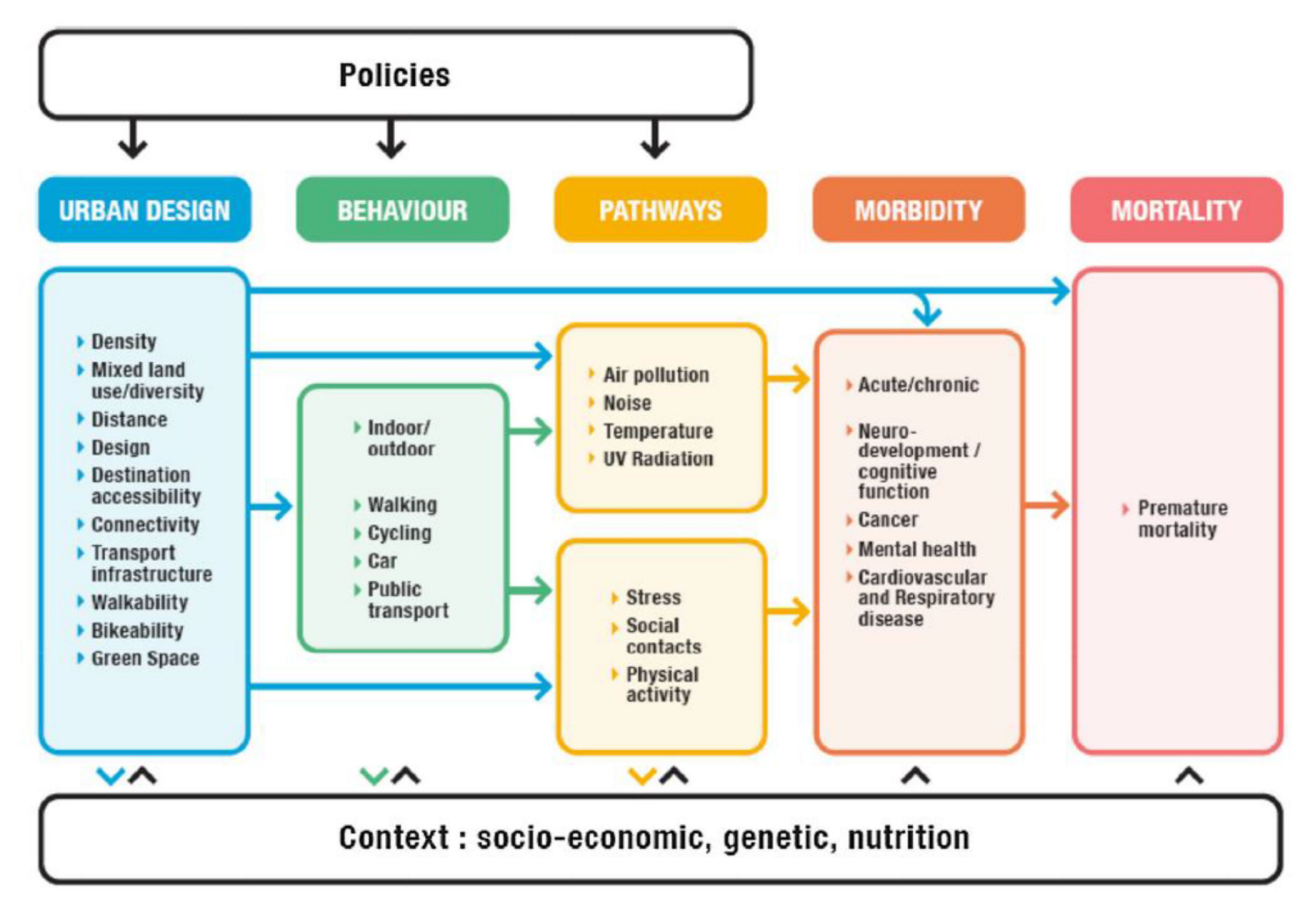
Conceptual framework of the links and pathways between urban design, environmental exposures and health^19^.

The pathways of urban form, environmental exposures, and health are intricate, and the magnitude of these associations have not been widely substantiated^18^. Although cities are a complex system, a conceptual framework developed by Nieuwenhuijsen & Khreis^19^ (Figure 1) illustrates the multitude of urban and transport planning pathways that contributes toward the health of urban populations. Urban form denotes the structure, design, and physical features of an urban environment^20^, captured by the urban design pillar in Figure 1. There are two dominant urban forms; the first, known as compact cities, is characterised by dense housing and road infrastructure, and the second by dispersed low density infrastructure with high sprawl^8,21^. Both are notionally inconducive to health and sustainability, as the first lends itself to increased pollutant emissions and noise levels, accentuated hot temperatures, and reduced green space^8^; whilst the second favours motorised traffic and motor vehicle dependency, poorer public transportation infrastructure, lower social cohesion, and reduced physical activity levels^4,22^. However, the compact city model has the conceptual benefits of shorter commuting distances that promote active mobility and increase social cohesion, which highlights the potential trade-offs and complexity of urban design^23^. Naturally, cities can be a combination of these forms.

The health burden attributable to environmental exposures in urban settings is well documented^7,8,24^. In 2019, particulate matter diameter 2.5μm (PM_2.5_) and ozone air pollution were estimated to cause 4.51 million premature deaths worldwide^25^, and road traffic injuries were ranked the leading cause of disability-adjusted life years (DALYs) for ages 10-49 years, ranking 10^th^ for ages 50-74 years^26^. Trends of increasing heat-related morbidity and mortality are largely ascribed to climate change^27^ and are exacerbated in urban environments due to the urban heat island (UHI) effect, an occurrence wherein urban areas exhibit elevated temperatures compared to their rural surroundings^28^. In addition to premature mortality, heat-related impacts include increased mental health distress^29^, cardiorespiratory-mortality^30^, and hospital admissions^31^. Although a lesser studied environmental risk factor, chronic exposure to noise pollution can also have adverse health effects; at least 20% of the European urban population is likely to be exposed to noise levels harmful to health^32^. In 2017, 18 million people in Europe were estimated to experience high annoyance from noise and 5 million sleep disturbance. Sedentary behaviour and reduced physical activity are well established risk factors of health burden and are often more prevalent in urban environments owing to lifestyles and built environment characteristics^22^. Perhaps the starkest of adverse impacts from sedentary behaviour^33^, sitting for 10 hours a day is associated with 48% increased risk of all-cause mortality compared to 7.5 hours a day^34^.

Translating health burden statistics into actionable recommendations for policy requires research to effectively discern the intricate relation between urban form, environmental stressors, and health. However, uncovering causal inferences is complex due to the multiple pathways, long causal chains, and dynamic nature of contextual factors (e.g., neighbourhood attributes) and compositional (e.g., demographic characteristics)^35^, alongside the multidisciplinary nature of urban and transport planning related impacts. Health impact assessment (HIA) is a widely adopted decision support tool that aids evidence-informed policies. HIAs are valuable within urban health research as the impacts of urban planning on health determinants and scenarios can be modelled and estimated impacts often have high comprehensibility to decision-makers, which helps generate awareness^36,37^. Temporal HIAs offer the additional advantage that predicted impacts reflect the historical trajectory of exposures and health burden, and thus, changes in exposure, impacts, and policies can be tracked over time^38^. To effectively interpret the accuracy of forecasted impacts and the existing evidence base necessitates understanding the uncertainties inherent in model assumptions and how these vary across studies^38^. Moreover, qualitative data, such as societal preferences, are integral in elucidating the constituents of an urban ecosystem. The Neighbourhood Environment Walkability Scale (NEWS) is one such tool designed to gather perceptions of neighbourhood attributes linked to physical activity (e.g., street connectivity)^39^. The widespread adoption of NEWS underscores the need for comprehensive, proxy tools that assess city liveability^40^. However there exists a plethora of different, context-specific walkability indices^41,42,43,44^; this underscores the resultant limitations in comparing studies that employ diverse methodologies, and the challenge in obtaining universally applicable insights into urban environmental health pathways and attributable impacts.

Large-scale urban studies offer generalisable and robust evidence for elucidating the nexus among city form, climate, transport, and environmental and health impacts. However, to the best of knowledge, there is no scoping review that synthesises evidence from large-scale urban studies that investigate these interconnections. Exploration of commonly employed methodologies, associated limitations, and key research gaps can highlight future research opportunities.

As such, the purpose of this scoping review was two-fold:

1) Synthesise evidence from large-scale urban studies that focused on the relation between urban structures, transport, environmental exposures, and health.
2) Advanced understanding of current knowledge and gaps, methodologies applied, limitations, and opportunities for the improvement of current research practice.

The research questions we sought to address were:

1) What methodologies were applied in urban form, transport and mobility, and urban environmental health studies from 2003 to 2023?
2) What are novel methods and indicators within urban environmental health research?
3) What knowledge gaps necessitate further exploration?

## 2 Methods

**Figure 2:**
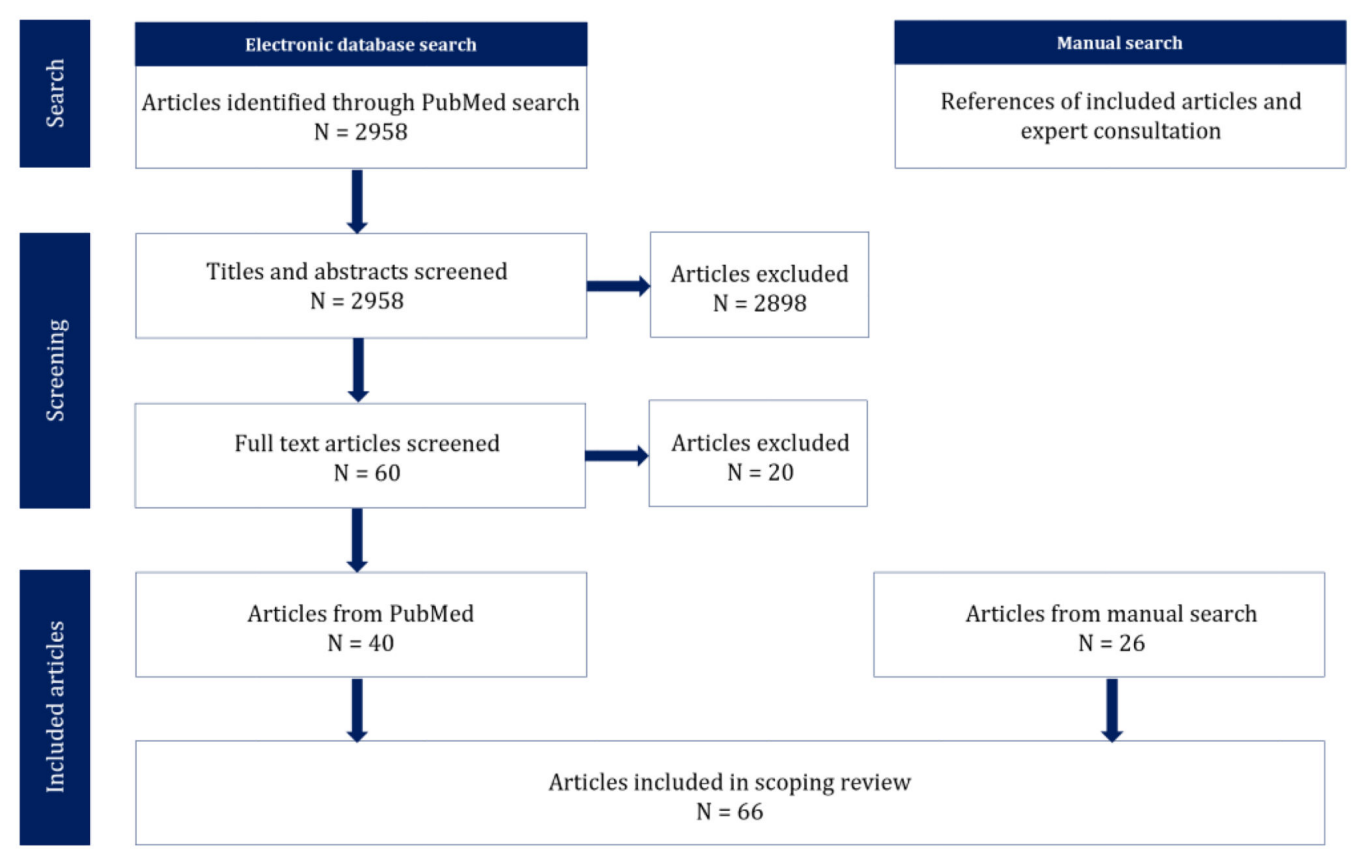
Flowchart of the literature search inclusion and exclusion process

This review was conducted as part of The Urban Burden of Disease Estimation for Policy Making project (UBDPolicy). UBDPolicy aims to improve the estimation of health impacts and socio-economic costs, or benefits, of environmental determinants in almost 1000 European cities in 31 countries^45^. Through provision of estimates of health impacts from air pollution^5^, noise^6^, heat^46^, and green space^47^ in regular three-yearly reporting intervals, UBDPolicy aims to advance understanding of wider impacts and trends from urban planning across Europe and build healthy and sustainable urban scenarios for specific case studies. Therefore, the conclusions drawn from this review and their applicability for UBDPolicy shaped the reasoning behind the methods employed. Given the exploratory nature required to meet the review’s objectives, we conducted a scoping review suited to identifying knowledge gaps and emerging methods within a broad topic area^48^. The anticipated heterogeneity of study designs of reviewed articles and practical and resource constraints rendered a systematic review or meta-analysis less suitable. Further, a UBDPolicy workshop held in Sitges, Spain, in October 2023 allowed expert consultation for identification of additional applicable studies. A literature search was performed using the bibliographical database PubMed. Figure 2 provides a visual representation of the process of article inclusion and exclusion.

### 2.1 Keywords search process

Seven independent searches using PubMed were carried out (Table 1). The same search terms to describe urban form were included in the seven searches. The first search focused on urban form and health, the second on urban environmental health, and the third on urban indicators. The distinction between urban form and urban environmental health pertains to the former investigating the direct link between urban form and health whereas for the latter, studies consider the exposure pathway either by assessment of urban form to environmental exposures or exposures to health.

**Figure 3:**
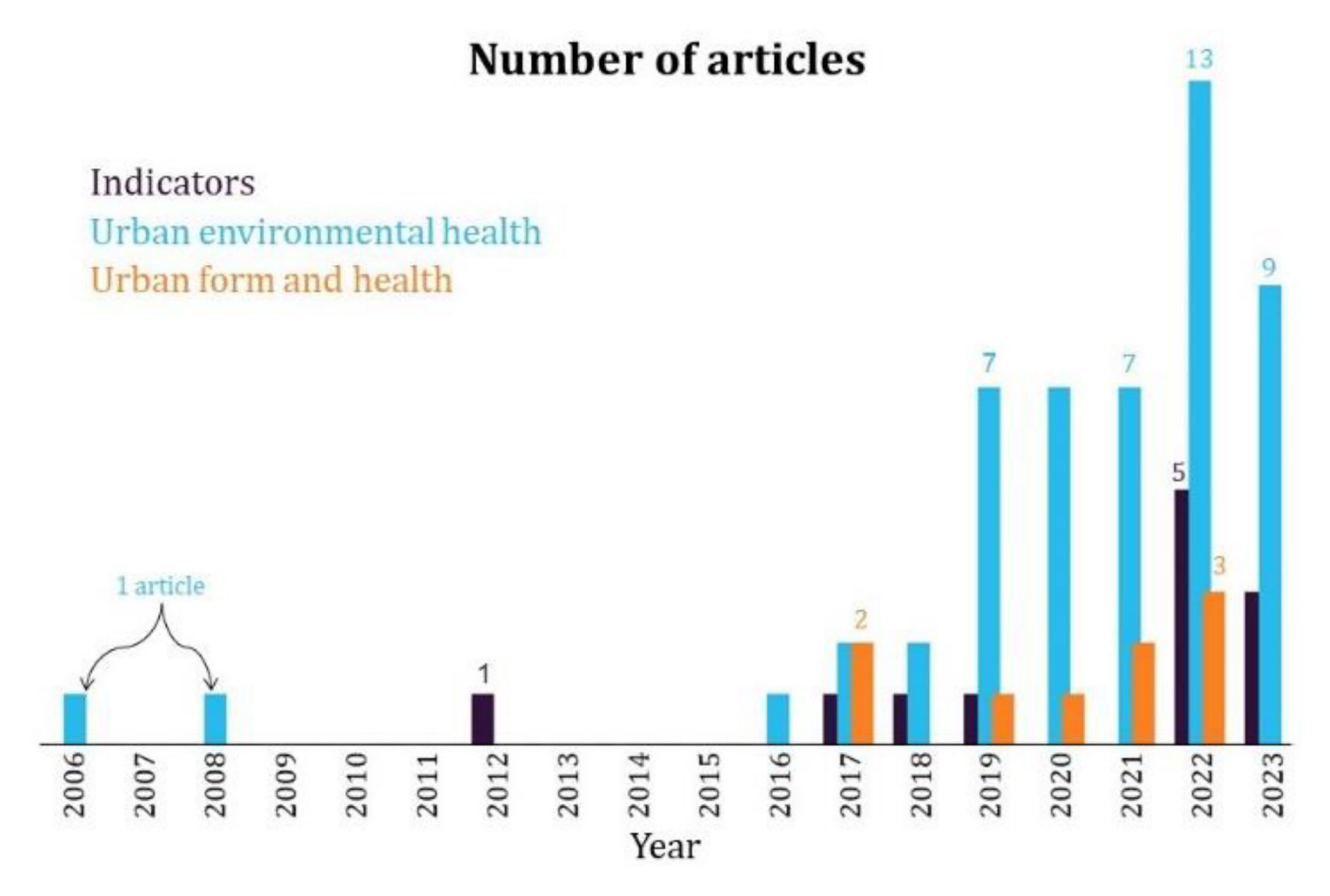
Number of articles by published year and theme.

For the second category of urban environmental health studies, five searches encompassed the following key themes: air pollution and health impacts; temperature and health impacts; green space and health impacts; noise and health impacts; and transport and mobility. The searches returned 2958 unique articles (Figure 2). Article abstracts were screened for relevance based on the inclusion criteria and objectives of UBDPolicy, which resulted in 40 papers for inclusion. An additional 26 papers were obtained from a manual search conducted by scanning reference lists for relevant studies and from expert consultation. This resulted in nine urban form and health studies 45 urban environmental health studies, and 12 urban indicator papers. A total of 66 studies were included. Table 1 provides a summary of the search terms used and results of each search. Figure 3 categorises articles by theme and year of publication.

### 2.2 Inclusion criteria

Article inclusion criteria and conducted searches were divided into three search categories; urban form and health, urban environmental health (subdivided into HIA studies and other research methodologies), and urban indicators. For the second search category, a distinction of HIA methodologies was made to allow for effective exploration of methodologies and affiliated challenges within the broader urban environmental health field. The inclusion criteria for search categories one and two (urban form and health and urban environmental health studies) constituted studies were required to have analysed at least 90 cities, be written in English, and published in peer-reviewed journals from January 2003 to December 2023. The inclusion criterion was set at 90 or more cities as this number was considered appropriate to standardise data collection across different environmental and climatic gradients and to be representative of studies with less than 90 cities. Studies published from January 2003 to December 2023 were included to ensure methodologies and findings were reflective of current levels of urbanisation and health impacts. For the second search category of urban environmental health studies, the environmental exposures included were: air pollution; temperature; green space; road traffic noise; and transport and mobility.

The third search category focused on urban indicators. Indicators and frameworks considered relevant were those that focused on urban design and environmental health. The inclusion criteria specified studies should be written in English and published in peer-reviewed journals from January 2003 to December 2023.

### 2.3 Exclusion criteria

The exclusion criteria applied to both searches encompassed environmental exposures not relevant to UBDPolicy (such as infectious diseases), studies that did not evaluate health impacts, health outcomes considered less attributable to city design and planning, and studies published before January 2003. For the second search category of urban indicators, the exclusion criterion of studies analysing less than 90 cities did not apply, as indicators can be scaled and applied to different contexts.

## 3 Results

**Figure 4:**
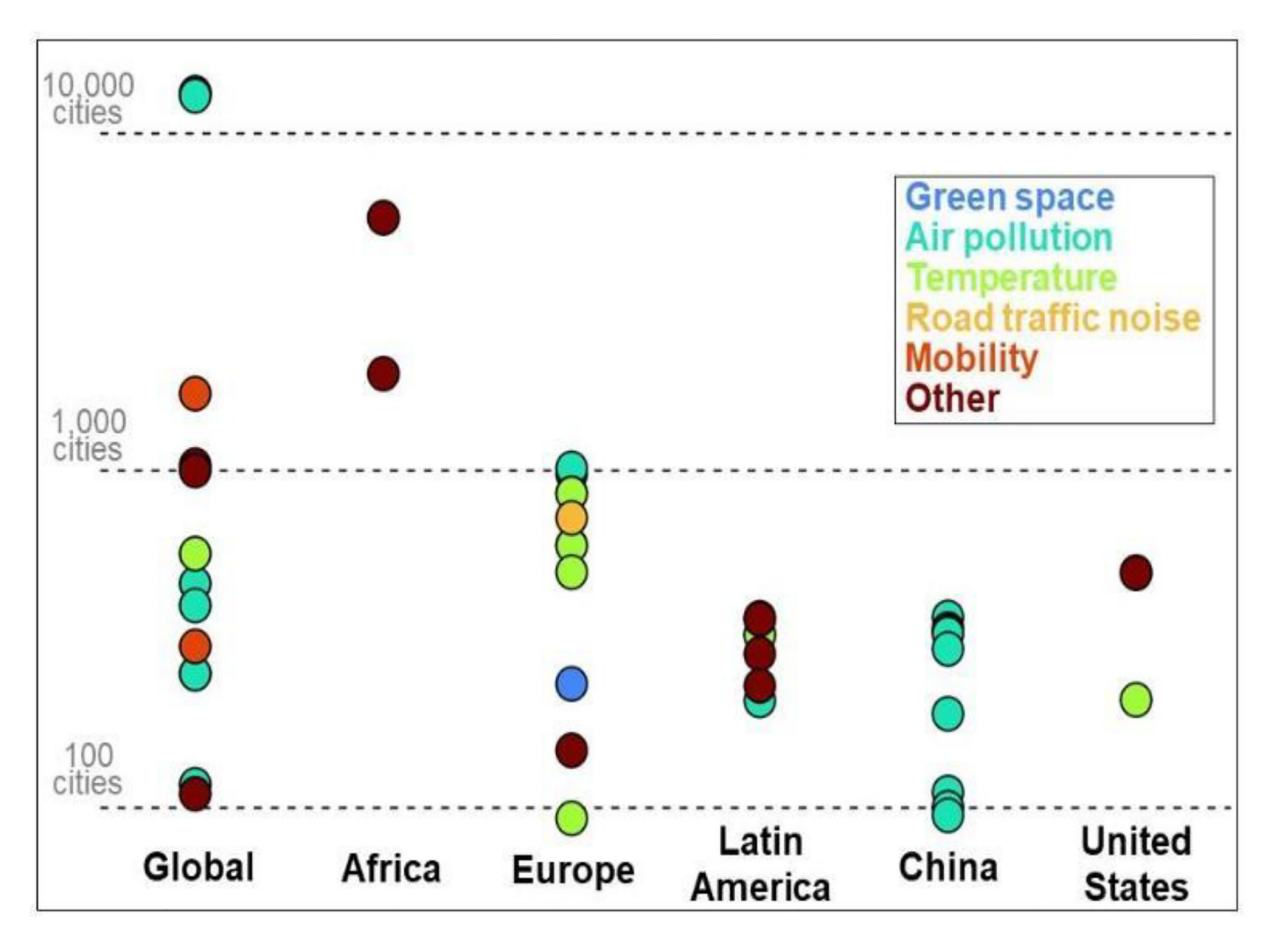
Number of cities analysed in each study, categorised by region and environmental exposure.

Of the 66 studies included in this review, the geographical regions covered were: Global (24), China (14), Europe (13), Latin America (9), the United States (3), and Africa (3) (Figure 4 and Table 2). While studies specific to South-Asia, South-East Asia, and the Middle East were not considered in this review, a number of cities from these regions featured in the global studies. A total of 45 studies examined urban environmental exposures and health, with the majority (29, ∼64%) assessing air pollution health impacts. The least studied exposure was road traffic noise (1, ∼1.5%). The number of cities analysed spanned a wide range (93 - 13,189 cities), with variation in city definitions employed (Tables 3 and 4). All studies conducted in China examined the health effects from air pollution exposure, whereas less studied regions, such as Africa were amongst the largest in scale in terms of the number of cities analysed (Figure 4). Examination of findings is in accordance with the thematic order outlined in Table 2, and constitutes four sections: urban form and health, urban environmental health, HIAs, and urban indicators.

### 3.1 Urban form and health

Many studies that assessed urban form employed urban form metrics at city-level, namely: population density^49,50,51,52^, fragmentation^53,50^, sprawl^21^, built-up area^53,21^, compact development^54^, intersection density^53^, and mass transit infrastructure^49,53^. Fewer studies explored spatial observations and patterns within-city level^52,54,55^.

Health outcomes included long-term and short-term outcomes; long-term outcomes encompassed non-communicable diseases, cancer-related mortality, infant mortality, and mental distress, whilst short-term outcomes were violence-related and unintentional injury-related mortality (Table 3). The only urban form studies to include social and demographic variables in analyses were conducted in Latin America and employed the social environment index, which comprises area-level measures of education attainment, access to water and sewage facilities, and overcrowding^50,53^. Higher values indicate more favourable social conditions and a higher quality of life.

Findings suggest that lower city fragmentation, high population density, high connectivity, and higher rates of public transportation have positive impacts on health and reducing premature mortality^49,53,55,56^. Car-centric urban planning^55^ was reported to have adverse effects on health, whilst in Africa greater sprawling cities were shown to have higher energy demands^52^. City size was identified as the most critical variable for influencing urban sprawl with round and compact city designs generally more advantageous^52^. Another African-based study conducted spatial analysis of four urban form variables in an effort to classify cities based on urbanisation dynamics^51^. Prieto-Curiel et al. developed a systematic approach to capture and delineate the spatial interactions between variables of city size, market potential, level of urbanisation, and local dominance; the latter indicates city size in relation to adjacent agglomerations^51^. Results showed diverse and distinct interactions of spatial variables, finding this to impact the rate of urban growth, the emergence of new agglomerations, and the clustering of cities. In another classification study, Taubenböck et al. utilised remote sensing and cluster analysis to classify 1500 cities worldwide into seven distinct types^54^. Findings highlighted the issue of spatial-morphological inequality, where the shape of cities was shown to be critical in shaping functional and social aspects of urban living, and 30% of sparsely built areas were found to accommodate 10% of the total population. Illustrating the complexity of urban form, a global study spanning 24 years found sprawl to strongly correlate with human development index (HDI), which comprises life expectancy, educational attainment, and standard of living (measured by gross national income (GNI) per capita); cities characterised by extensive urban sprawl exhibited high values of HDI^21^. Between 1990 and 2014, Europe was identified as the continent with the highest degree of urban sprawl and had the highest sprawl rate, increasing by 51% since 1990^21^.

### 3.2 Urban environmental health

Urban studies that investigated the exposure pathway to health in general followed an ecological (10, ∼15%) or cross-sectional study design (6, ∼9%), with a minority encompassing modelling studies (2, ∼3%), or meta-analysis (1, ∼1.5%) (Table 3). Certain studies adjusted for population demographic characteristics in their analyses, such as household income^57^, income inequality^58^, self-rated health^55^, educational attainment^49^, and race and ethnicity^57^. Seven studies (∼11%) directly examined the modification effect of socioeconomic status (SES) on the association between the urban environment and health, applying gross-domestic product (GDP) per capita^59,60,61^, GINI coefficient^58,61^, or GNI per capita^62^. In all studies that performed stratified analyses of socioeconomic (SE) and demographics variables, aggregate data were applied at city-level.

#### 3.2.1 Urban form and air pollution

Studies consistently reported significant proportions of urban populations to be exposed to ambient pollution that exceeded WHO 2005^10,59,63,64^ and 2021^60,65^ guidelines. Findings from Latin America showed 85% of the study population exposed to ambient nitrogen dioxide (NO_2_) concentrations and 58% exposed to PM_2.5_ levels that exceeded WHO guidelines^59,60,^. Whilst Anderson et al. reported all the 5625 African cities under study failed to meet WHO 2005 clean air guidelines^10^.

The relation between city size, higher population density, and pollutant concentrations was somewhat inconsistent. A Latin American study reported larger population size was associated with higher annual mean PM_2.5_, whilst higher population density was positively associated with lower levels of PM_2.5_ in a separate univariate model^59^. Another Latin American study reported denser and more congested cities to have higher NO_2_ and PM_2.5_ concentrations, owing to higher motorisation rates and congestion^60^. The same study reported highest variability in NO_2_ population exposure was within cities and an increase in green space at neighbourhood level, rather than city-level, was associated with lower local levels of NO_2_^60^. Interestingly, Rezaei & Millard-Ball observed cities with greater density exhibited reduced per capita PM_2.5_ transportation emissions; however, increased exposure was noted due to the population residing in closer proximity to emission sources^62^. Authors noted greater variation in emission exposure between income groups, as opposed to urban form metrics and income where no significant correlations were found. Another study found higher city GDP per capita and higher intersection density correlated with elevated levels of PM_2.5_^59^. The only study to include educational attainment in analyses found population groups of higher educational attainment were exposed to higher NO_2_ concentrations^60^.

### 3.2.2 Urban form and temperature

Studies that assessed the relationship between urban form, temperature, and health mainly focused on the impact of non-optimal temperatures on premature and cardiovascular-related mortality^58,66,67^. In Europe, lower minimum mortality temperature (MMT) positively correlated with lower GDP per capita; for example, spatially close cities of Austria (Vienna) and Slovakia (Bratislava) exhibited MMTs of 20.5°C and 18.4°C and GDP per capita of 29,301 and 11,348, respectively^61^. A Latin American study found the GINI coefficient, indicative of income inequality, was the sole modifier that showed a statistically significant association with all-age MMT^58^. Cities exhibiting the highest income inequality experienced a mortality rate 3.45% higher than those in the lowest tertile of income inequality^58^. For ages 65 years and older, increased levels of poverty and residential segregation were linked to higher cold MMT^58^. Of note, there were higher deaths associated with cold, 5.09% out of 5.75% non-optimal temperature attributable deaths at all ages, compared to 0.67% deaths associated with heat^58^. Zhou et al. found city size and compactness to have the strongest influence on UHI intensities, concluding small to medium sized cities were most effective in alleviating UHI^68^.

#### 3.2.3 Urban form and green space

Generally, studies found the health benefits of urban green space to depend upon the distribution within a city^57,62,69^. Reported health benefits included lower levels of obesity^57,69^, mental health disorders^57^, and lower pollutant levels^10,67^. Across African cities, linear econometric models predicted the impact of increasing green space cover by at least 25% and found this would reduce PM_2.5_ to moderately safe levels (12 - 35.4μg/m^3^)^10^. Evidence varied on whether the type of green space had an effect on benefits. Olsen et al. explored a range of land uses and the impacts at individual and aggregate city-level across European cities and found relatively wild green space (constituting agricultural, wetlands, and semi-natural areas) was associated with lower standardised mortality rate^70^. Another study found a significant correlation between poor mental health and greenness and between obesity and tree cover, reporting no significant relationships between greenness and obesity, or between tree cover and mental health^57^. A notable strength of Browning et al.’s study was the inclusion of moderation tests for exploring effect modification, analysing sociodemographic variables and urban sprawl (defined by population density, the percentage who drive to work, and residential density). When adjusting for spatial and confounding variables, population density (-0.15, -0.17), physical inactivity (0.65, 0.67), median age (-0.11, -0.11), and income (-0.98, -0.95) were significantly associated with obesity (reported β coefficients are for greenness and tree cover, respectively). Whilst median income (-0.85, -0.86) and physical inactivity (0.21, 0.2) were significantly associated with poor mental health^57^.

Although evidence was mixed, urban form characteristics of denser housing^70^, higher population density^71^, and more compact cities^10^ generally showed a negative association with green space availability. Aiming to advance predictions of the benefits of increasing green space, Marando et al. developed a model that simulated the microclimate regulation of urban green infrastructure across European cities^72^. To lower temperatures by 1°C in urban areas, a minimum tree cover of 16% was required. Of the Functional Urban Areas (FUAs) studied in Europe, 32% (192 FUAs) had tree cover below 16%. A global review by McDonald et al. explored how urban areas can achieve both population density and green space and found a 10% increase in density was associated with 2.9% decline in tree cover^72^. Interestingly, the reported negative correlation was weakest when explored at neighbourhood level compared to city-level, suggesting some neighbourhoods achieved more tree canopy than was expected based on population density. Supportive findings by Anderson et al. observed variation between cities in the magnitude of cooling benefits from green space and attributed this to different distributions of green space within cities^10^. Cities with the same availability of green space (20%) but different levels of proximity experienced varying cooling effects during a heat wave, 55% of one city’s population was estimated to benefit in contrast to 16% of another city’s population^10^.

#### 3.2.4 Urban form and transport and mobility

Bassolas et al. developed a metric that quantifies the hierarchical organisation of urban mobility, considered a proxy for urban inhabitants’ needs being met^73^ (Table A1 in Appendix). Weekly trip flow information of 300 million people in 301 global cities was aggregated into weighted networks to identify hotspots of activity at spatial resolution of ∼1.27km^2^ and city-level. The varied spatial distribution patterns of hotspots captured differences in city organisation, permitting inferences of the effects of urban structure on transportation (mode share), pollutant emissions, and health outcomes (ischaemic stroke mortality and fatal traffic injuries). Greater urban mobility was attributed to more population mixing (Pearson’s coefficient (R^2^_P_) = 0.21, Spearman’s coefficient (R^2^_S_) = 0.24), extensive use of public transportation (R^2^_P_ = 0.45, R^2^_S_ = 0.39), higher levels of walkability (R^2^_P_ = 0.47, R^2^_S_ = 0.58), and better health outcomes (ischaemic stroke mortality rate per 100,000 inhabitants: R^2^_P_ = 0.31, R^2^_S_ = 0.26, fatal traffic injuries: R^2^_P_ = 0.34 and R^2^_S_ = 0.33). Another study that applied advanced techniques of remote sensing and global geospatial data identified nine global city types by modularity analysis^74^. The poorest performing cities for road traffic injuries were characterised by sparse and irregular shapes with large blocks, whereas the best performing city types were characterised by high rates of public transportation. Road traffic injury burden of 9.6 million DALYs were attributed to suboptimal urban design^74^.

### 3.3 Health impact assessment

Of the 45 urban environmental health studies, 25 applied a HIA methodology. All the HIAs followed a comparative risk assessment (CRA) approach, with all but one HIA^121^ assessing the potential health impacts under an alternative scenario (i.e., counterfactual)^38^. To effectively examine the different HIA methodologies employed, this section is structured as follows: environmental exposures, population and health data, exposure response functions (ERFs) and counterfactual scenarios, and summary of findings.

#### 3.3.1 Environmental exposures

Almost 85% of the HIAs (21) analysed the health impacts from air pollution. Of these HIAs, eight obtained pollution exposure data from the common data repository of China National Environmental Monitoring Centre^122^, two utilised a dataset produced by Anenberg et al.^123^, and the remainder obtained estimates from emission inventories^124,125,126,127,128,129,130^ or from air pollution models (e.g., land use regression models, EMEP MSC-W chemical transport model, and SHERPA tool)^5,131,132,133^ (Table 4). The majority of HIAs that focused on air pollution analysed PM_2.5_ as the environmental exposure (14, ∼56%), followed by ozone (8, ∼32%), NO_2_ (7, 28%) and particulate matter diameter 10μm (PM_10_) (2, 8%) with one study assessing carbon dioxide (CO_2_)^131^ and one sulphur dioxide (SO_2_) and total suspended particles (TSP)^134^. Of the 25 HIAs, eight (32%) assessed temporal trends in air pollution, the longest trend assessed global NO_2_-attributable paediatric asthma incidence across 29 years^123^.

Of the four HIAs that analysed alternative environmental exposures, two assessed temperature health impacts^48,135^, obtaining temperature records from ERA5-Land dataset (100m^2^)^48^ and Copernicus UrbClim model application (100m^2^)^135^; one assessed green space^49^ by normalised differential vegetation index (NDVI) and percentage of green area (%GA), obtained from the US Geological Survey^89^ and European Urban Atlas^136^ (250m^2^); and one estimated the impact of road traffic noise^6^. Of the strategic noise maps acquired from the Environmental Noise Directive and local sources ∼83% were considered low or moderate quality. Masselot et al. was the only HIA to analyse both extreme heat and extreme cold^115^.

#### 3.3.2 Population and health data

Similar city population data sources were applied based on the country HIAs were conducted in. For HIAs conducted in China, the National Bureau of Statistics of China was a common population data depository; all HIAs conducted in Europe (6, 24%) utilised the Urban Audit, whilst Global HIAs obtained population estimates from European Commission’s Joint Research Centre or the Centre for International Earth Science Information Network (CIESIN) (Table 4). Health data were generally obtained at national or provincial-level and applied to city-level; two HIAs in China^128,134^ and all HIAs conducted in Europe utilised city-level health data.

A diverse range of health outcomes were analysed, with each HIA examining between one and 24 health outcomes (Table 4). Mortality outcomes were a key focus, encompassing categories of all-cause mortality (14, 56%), cause-specific mortality (8, 32%), natural-cause mortality (3, 12%), and specific morbidity-related mortality (6, 24%). Mortality estimates mostly obtained from the Global Burden of Disease study^137^. Units ranged from total death counts, mortality rate per 100,000, DALYs and Years of Life Lost. Beyond morbidity and mortality, additional health outcomes included attributable hospital admissions, symptom onset, and high noise annoyance^130,6^. Notably, the majority of HIAs assessed health impacts in adults. Only two HIAs (8%) assessed health outcomes in children, focusing on premature paediatric mortality^123^ and asthma attack, respiratory symptoms, and bronchodilator usage^132^.

#### 3.3.3 Exposure response functions and counterfactual scenarios

The most common sources of ERF were from epidemiological literature. Two HIAs obtained ERF estimates from local cohort studies, whilst one HIA estimated ERFs by atmospheric modelling with integrated risk function based on six meta-analyses^129^. Only one HIA developed their own ERFs^121^, and these were applied in another HIA to estimate UHI impacts^46^. Masselot et al. employed a three-stage modelling framework that applied daily time series temperature and mortality data, age-specific mortality, and composite indices of vulnerability to produce age- and city-specific ERFs^121^. The composite index of vulnerability was developed from distributed lag non-linear and meta-regression models and incorporated city size, proximity to green and blue space, and SE inequalities^121^. In general, ERFs were applied homogeneously to the adult study population. Exceptions included acute lower respiratory infection-specific ERF to infants under five years^132^, city-specific and age group-specific ERFs for temperature^46,121^, and morbidity- and health endpoint-specific ERFs^132,138,127,130^. There was variation in counterfactuals applied. Of the 13 HIAs (25%) that analysed health risk of PM_2.5_ exposure, five applied the same counterfactual 10μg/m^3^ based on the 2005 WHO guideline, whilst three applied the 2021 guideline of 5μg/m^3^^126,128,129^. For air pollution, counterfactuals ranged: for PM_2.5_ 2.4 - 35μg/m^3 126,139^; ozone 54 - 160μg/m^3 139,140^; NO_2_ ∼3.78 - 80μg/m^3^ and PM_10_ 5.8 - 40μg/m^3^^130,132^. Two studies applied Chinese ambient air quality standards (CAAQS) as counterfactual scenarios^130,134^, whereas Khomenko et al.’s study was the only one to apply the lowest measured concentration in the dataset as an additional counterfactual concentration^5^. Barboza et al. based counterfactuals on the WHO recommendation of universal access to green space (i.e., equal opportunity to access) within 300 m of residence, applying counterfactuals of 25% GA within 300m of residence and a target NDVI modelled for each city^47^. Another HIA based in Europe estimated the mortality burden attributable to UHI by applying city-specific counterfactuals of exposure level scenarios without an UHI effect and estimated the impact on mortality by increasing tree coverage to 25%, 30%, and 40%^131^. The only study to focus on road traffic noise health impacts applied WHO recommendation of 53dB, which remains the current guideline^6^.

#### 3.3.4 Summary of findings

Global HIAs consistently reported cities in southeast Asian countries to experience the greatest pollutant concentrations and attributable health impacts worldwide^129,131,141,142^. Inconsistent findings from HIAs conducted across the same years 2015 and 2020 in China reported ozone-related impacts increased by ∼95% (5.05 x10^6^ DALYs) and 96% (7.64 x10^5^ DALYs) for all-cause and respiratory mortality^139^, respectively, in contrast to ozone-attributable impacts reported to increase by 17% for all-cause mortality (133,415 deaths in 2015 to 156,173 deaths in 2020) and 17% for respiratory mortality (28,614 deaths in 2015 to 33,456 deaths in 2020). For NO_2_, a global HIA reported highest NO_2_-attributable deaths in South Asia (75,397 deaths) and Eastern Europe (46,840 deaths)^142^. Whereas within Europe, Khomenko et al. reported the highest NO_2_ mortality burden was in Western and Southern European capital cities and applied local-level mortality rates; highest burden cities were Madrid (Spain), Antwerp (Belgium), and Turin (Italy)^5^.

Temporal trend HIAs revealed declining trends in PM_2.5_ concentrations and attributable mortality in China and globally^126,128^. Southerland et al. reported the largest absolute decrease in mean urban population-weighted PM_2.5_ concentration between 2000 and 2019 was in Africa, decreasing by 18%^129^. However, in certain regions, such as Luanda (Angola), there was an increase in PM_2.5_ concentrations and directional trends did not consistently align with trends in attributable mortality rates (an observation potentially explained by reported population growth). Another global temporal HIA covering 2000-2019 reported South and East Asia accounted for the highest proportion of global population ozone-attributable mortality in 2019, followed by Eastern Europe. However, this HIA reported divergent trends within South and East Asia; population-weighted ozone concentrations and mortality rates increased across all cities in South Asia, and decreased across all cities in East Asia^141^.

Additional insights from temporal trend analyses were the contribution of HIA parameters to health impact estimates. For ozone-attributed mortality, key global drivers were ozone concentrations and population, and for a few regions changes in baseline disease rates^141^. For PM_2.5_-attributed mortality, changes in population growth and population ageing were the primary drivers in all regions^129^. For specific cities across Africa, the Eastern Mediterranean, and Southeast Asia, changes in baseline disease rates had the largest impact. Conversely, in the Western Pacific, the Americas, and Europe, reductions in PM_2.5_ concentrations outweighed the influence of baseline disease rates^129^.

In addition to regional variation in exposure attributable health burden, there was heterogeneity among cities and age groups. In Europe, cities in Northern Italy were amongst cities with the highest mortality burden despite Italy not placing highest for PM_2.5_-attributed mortality burden in country-level estimates^5^. Similarly in Europe, Barboza et al. reported 42,698 and 17,947 annual deaths could be prevented by increasing NDVI and %GA, respectively, and found unequal distribution of NDVI and %GA among and within cities^47^. The only HIA to assess the impacts of non-optimal temperatures reported large variability in vulnerability across Europe^121^. The highest vulnerability was found in eastern European cities during extreme cold and heat and in age groups of over 85 years, which contributed over 60% to the total mortality burden. Annual excess deaths of 203,620 deaths (129 per 100,000 person years) were attributed to cold temperatures and 20,173 annual excess deaths (13 per 100,000 person years) attributed to heat. Iungman et al. found that increasing tree coverage to 30% can reduce city temperatures by 0.4°C and prevent almost 40% (2644 premature deaths) of 6700 premature UHI-attributable deaths^46^. The only study to examine the effects of noise on health reported 11 million adults, of the estimated 60 million exposed to road traffic noise, to experience significant annoyance and 3608 IHD-deaths could have been prevented if compliance with WHO recommendations were achieved^6^. City comparative analysis was not possible due to inconsistencies in noise mapping methods.

### 3.4 Indicators

Identified indicators covered the key themes of this review: urban form, air pollution, temperature, green space, noise, and transport and mobility; in addition to climate change mitigation, which encompassed indicators of greenhouse gas emissions and climate change impact on trees. The indicators identified and methods employed, in addition to geographical coverage, spatial resolution, and data sources, are detailed in Table A1 of the Appendix. There was heterogeneity in spatial resolution of indicators; the greatest variation was amongst air pollution indicators, which ranged from 0.01° resolution to the coarsest resolution of NUTS3 level, a territorial unit defined by the European Commission Urban Audit that typically encompasses districts or boroughs^203^ (Table A1).

As part of a *Lancet* series on urban design, transport and health^204^, Boeing et al. developed an open-source framework with urban spatial indicators for measuring walkability and public transport access^205^. A total of 25 global cities were compared to elucidate the optimal urban design for promoting active travel^35^. Applying the developed walkability index, Boeing et al. found compact cities had better walkability, whereas the worst performing cities for active travel were concentrated in more sprawled cities in high-income countries (HIC), such as Australia and the United States, consistent with previous findings^21,206^. To add to the utility of these indicators, Cerin et al. sought to provide evidence-informed thresholds^207^. To meet the physical activity criteria of urban inhabitants having at least 80% probability of engaging in walking for transport, and WHO’s target of at least 15% relative reduction in insufficient physical activity through walking^208^, neighbourhood targets associated with meeting one or both criteria were identified as: 5700 people per km^2^, 100 intersections per km^2^, and 25 public transport stops per km^2^. Curvilinear associations of population, street intersection, and public transport densities with walking revealed less than a quarter of the studied population lived in neighbourhoods that reached these thresholds, with observed between-city differences; cities in Latin American upper-middle-income countries performed better than those in HIC. Another transport and mobility indicator that aimed to measure how conducive the urban environment is to active transport was the extent of bicycle network in a city^209^. Akande et al. utilised the UNECE-ITU Smart Sustainable Cities Framework to rank 28 European capital cities based on 32 sustainability indicators covering the thematic areas of economy, environment, and society and culture^210^. Berlin (Germany) was ranked the most smart and sustainable city; indicators of bicycle network, wastewater treatment, and e-commerce had the greatest impact on ranking. Conversely, Sofia (Bulgaria) and Bucharest (Romania) were the lowest ranked cities, rankings were most influenced by indicators PM_10_ emissions and protected terrestrial area (Table A1). Other novel indicators of urban form included access to urban services and amenities, considered proxies for opportunities and living standards within cities^211,205^.

Climate change mitigation indicators have the potential to advance understanding of how cities contribute to climate change, forecast impacts, and potential mitigation strategies. One indicator depicted the percentage change in greenhouse gas emissions between 2000 and 2020 at city-level, disaggregated by pollutant and sector (e.g., agriculture from livestock, soils, and waste burning, industry, residential, commercial, and off- and on-road transportation)^211^; in addition to a 20-year global warming potential and total emission summaries for 2000 and 2020 (Table A1). Pertinent to climate change urban mitigation strategies, the average annual greenhouse gas net flux from trees (per hectare of city area) was provided for a 21-year period, 2000 to 2021 (Table A1). This is complimented by an indicator of the same global coverage, which estimated the percentage of urban built-up land absent of tree cover^211^. Related temperature indicators included the percentage of built-up land with low surface reflectivity^211^. This enables identification of areas within a city that exhibit low solar reflectivity and thereby could derive significant benefit from the implementation of tree planting and green spaces.

Departing from commonly applied green space indicators that measure NDVI and %GA, novel methods for analysing green space included accessibility, quality, level of urban biodiversity, and the relation between green space and inequality (Table A1). Battiston & Schifanella developed a composite index for green space accessibility and exposed variation between-city levels; cities in Europe and Australia-Oceania had higher green space accessibility compared to regions in low- and middle-income countries and North America^212^. The index’ sensitivity to parameterisation was evident from adjustment of metrics, such as level of inequality (defined by the GINI coefficient), resulting in different area rankings of green space accessibility. Complimentary work has aimed to quantify green space accessibility based on quality, defined as “high-amenity nature”^213^. Ranking cities by amenity of accessible nature revealed higher population densities, although living generally further from nature, live closer to high-amenity nature compared to residents of lower urban population densities. Further advances for analysing green space were illustrated by Stowell et al. who applied cloud computing technology and analysis of remote sensing data to produce an urban greenness indicator dataset (measured by population-weighted peak and annual mean NDVI). Although an NDVI metric is not novel, 1000 global cities were classified based on level of greenness, climate zone, and HDI for the years of 2010, 2015, and 2020, which allows for temporal tracking of urban greenness– an attribute not available in other reviewed indicators^214^ (Table A1).

## 4 Discussion

The purpose of this review was to synthesise evidence from large-scale urban studies that focused on the relation between urban structures, environmental exposures, and health and to identify future opportunities for urban health research. To achieve this, the research questions we sought to address were: what methodologies were applied in urban form, transport and mobility, and urban environmental health studies from 2003 to 2023? What are novel methods and indicators within urban environmental health research? What knowledge gaps necessitate further exploration?

Key findings from this review confirm the complex, intricate relation between the urban environment and health. This is evidenced from the discordant impacts from urban form variables on exposures and health. For example, compactness^52,54^, high population density^49,50,51,52^, green space^57,62,69,47^, and extensive public transportation and active travel infrastructure^49,53,73,207^ were found to have a multitude of benefits, which promote health and well-being^73,205,207^. Conversely, increasing density and compactness were associated with the trade-offs of reduced green space^10,71^, accentuated UHI^46,68^, and higher pollutant concentrations and exposure from congestion^59,60^. Urban sprawl and fragmented city shapes were generally reported to have negative implications for city liveability^54^ and health^50,53^. This pertains to the ‘15-minute city’ model, wherein all essential amenities for the urban residents’ needs, such as health, socialisation and culture, are accessible by walking or cycling within a 15-minute radius^215^. The strong correlation between urban sprawl and HDI could indicate sprawl has positive ramifications, owed to HDI incorporating life expectancy, educational attainment, and gross national income per capita^21^. Urban scaling laws offer a partial explanation, as linear urban scaling delineates that larger cities generate higher wages^216^, consistent with findings of city size being the most influencing factor for urban sprawl^52^. Spatial analysis of urban form characteristics by Prieto-Curiel et al. demonstrated concomitant analysis is critical for understanding how urban shape and structures affect the functional and social aspects of urban living^51^.

An important inference from reviewed literature is the distinction between exposure and vulnerability, as certain less-exposed groups may have heightened vulnerability to the exposure under study. For example, sophisticated methods employed by Masselot et al. found the highest vulnerability to extreme cold and heat was in age groups of over 85 years^121^. Differential risk levels from extreme temperatures based on gender have been illustrated elsewhere, women aged 65 years and above and men below 65 years showed the highest vulnerability to hot temperatures^217^. In Europe, groups of lower SES had lower MMT^61^, whilst in Latin America higher levels of poverty and income inequality were associated with all-age MMT and higher cold MMT^58^. Inequality-driven variation in exposure levels was also present; reduced access to green space and therefore increased PM_2.5_-exposure was reported in lower income groups^62^.

### 4.1 What methodologies were applied in urban form, transport and mobility, and urban environmental health studies from 2003 to 2023?

There was heterogeneity across studies in methodologies, indicators, and city boundaries (Table 3 and 4). Sub-city units can vary in size and composition, and therefore, the boundaries of urban agglomerations can have a considerable effect on results, creating a potential bias towards larger cities^10^. Harmonised city definitions are a key challenge and may have contributed to contrasting results. To achieve cooling effects of urban green in Europe, tree cover of at least 16% was estimated to achieve a reduction of 1°C^72^, whilst an HIA study estimated 30% tree cover would be required to reduce temperatures by 0.4°C^46^. Iungman et al. employed a city-level model^46^, whilst Marando et al. utilised FUAs^72^, which encompass the surrounding community zone and suburban areas^218^. Approaches to defining cities of the reviewed studies were based upon administrative boundaries^5^, functional definitions that rely on travel patterns and economic connections^72^, or morphological approaches that create shapes based on the extent of built-up or urbanised areas^62^; the choice of definition typically depends upon research objectives. An operational city definition independent of context specificity would improve meaningful comparisons and transparency among studies.

The prevailing study design applied was cross-sectional or ecological (Table 3), which reflects a wider challenge in the field of requiring longitudinal studies and thus more robust causal inferences of the relation between urban design and health^219^. This has further implications that the exposure-response relationships may be limited and therefore captured in analyses. For example, the link between urban land use, transport and mortality, and health is conceptually well understood; however, it lacks comprehensive quantitative evidence^15^.

In addition, the exposures under study may not fully capture population exposure. In urban environmental health studies focused on green space, proximity was the primary exposure variable analysed. Exploration of the frequency^220^ that urban residents visit green space, potential variation in access between demographic subgroups^220^, and the quality and amenity can augment the understanding of population exposure and attributable health impacts. Research examining spatial inequalities in quality and accessibility of green space consistently report residents of more deprived neighbourhoods experience longer travel time to access green areas^221,222^. In Brussels (Belgium), area-based deprivation levels were associated with reduced satisfaction and authors identified factors that influence the use of green space, such as positive attributes of tranquillity and cleanliness and negative attributes of noise and lack of facilities^221^. Further, none of the reviewed air pollutant studies explored indoor air pollution. Although often present at low concentrations, long-term exposure to indoor air pollutants can pose significant risk to human health^223^. Given that people spend the majority of their time indoors, incorporation of indoor pollutant exposure estimates would ensure predicted health impacts are comprehensive and effectively advance the understanding of the magnitude of this exposure pathway. Novel materials for sensors, indoor air pollution-monitoring systems, and smart homes show promise for advancing exposure and impact estimations of indoor air quality^223^.

In comparison to the other study designs employed, the HIA methodology can present distinct advantages; however, equally have distinct challenges. Within China, divergent estimates of ozone-attributable impacts for all-cause and respiratory mortality highlight the sensitivity of methodological choices^139,140^. These respective studies applied the largest difference in counterfactuals of pollutant HIAs reviewed; Guan et al.^139^ estimated impacts relative to 160μg/m^3^ whereas Zhang et al.^140^ applied counterfactual of 54μg/m^3^. This may partially explain varied findings and highlights the significance of counterfactual scenario choices, in addition to the difficulty in study comparisons when different health outcomes are assessed (e.g., DALYs *vs*. deaths). Further, models used to calculate pollutant exposure levels are generally built using data representative of the average exposure and thus extremes in concentration response relationships are poorly understood. Investigation on the significance and choice of counterfactual scenarios was beyond the scope of this review; however, it highlights an important conjecture when conducting HIAs and interpreting results.

Additional insights from temporal trend HIAs were the ability to track impact over time and identify impact drivers of policies and exposure level changes. This can introduce the methodological challenge of the sensitivity ascribed to chosen years. Of the eight temporal studies, three included the year 2020 and thus the COVID-19 pandemic is likely to have influenced exposure levels and impact estimates^139,140,189^. Whilst estimates of temperature-attributed health impact will be largely affected by a particularly hot year being included in analyses. Advances in available indicators that permit temporal tracking will improve the accuracy of temporal estimates and help mitigate this constraint. The only identified indicator that included temporal tracking was for green space availability, which may be particularly useful in understanding climate change resilience of different urban green types^214^.

### 4.2 What are novel methods and indicators within urban environmental health research?

The importance of studying local variance of environmental exposures and health impacts was illustrated and new methods and indicators show promise to this advancement. African cities with the same availability of green space were found to experience varying cooling effects during heat waves^10^. This was ascribed to varied distributions of green space within cities, suggesting availability is not the same as proximity and quality. This inference was corroborated by Barboza et al. whose sensitivity analyses suggested population distribution within cities influenced local differences of green space-attributable health impacts^47^. To achieve a balance of dense and green cities, future research analysing the cooling effects of urban tree cover should consider the effects of climate change and urban green resilience^224^. The greatest environmental benefits are considered to be provided by long-stature, mature trees and thus this is an important consideration for the time required and potential impact of climate change and UHI mitigation strategies^224^. Novel green space indicators of green space quality^213^, level of amenity^209^, and urban biodiversity^211^ offer to advance this understanding. The latter may improve understanding of the ecological quality and species-richness; greater biodiversity closer to residence requires large urban connected patches and offers positive benefits on mental health and well-being^10^.

The emergence of cutting-edge technologies^225,226^ and advances in remote sensing and geospatial data sources present significant opportunities to enhance the comprehension of intricate urban health phenomena and the identification of key elements for sustainable urban design^47,219^. These advancements hold the potential to address challenges related to diverse urban form metrics and definitions by leveraging geospatial data sources. These sources can improve the accuracy of population-weighted averages for obtaining overall urban metrics or enable the disaggregation of cities into neighbourhoods, thus facilitating better harmonisation. A key challenge will be effective translation of vast quantities of remote sensing and other spatial data sources into interpretable evidence of the complex spatial interactions^219^; however, deep learning algorithms offer a promising solution to this challenge, through techniques such as semantic segmentation^227^.

Further applications of spatial data science and artificial-intelligent (AI)-driven tools for supporting sustainable urban development include agent-based modelling (ABM)^228^ and machine learning algorithms^225^. Motieyan et al. utilised an ABM to simulate the implementation of superblocks, an urban model that prioritises public space for active transport and leisure and minimises motorised traffic^229^. By incorporating individual “agents” diverse behavioural patterns of local citizens were simulated which enabled anticipation of public opinion and acceptance of superblock implementation. Machine learning algorithms are enhancing predictions of environmental exposures, through methods such as integration of urban morphology data (e.g., topography and building height) into air quality forecasts^230^. Woo Oh et al. trained deep learning models using meteorological data and urban texture factors (e.g., surface albedo) to develop temporal- and spatial-UHI models^231^. The temporal UHI model that quantified the number of UHI hours rather than intensity, was found to be a better predictor of seasonal UHI predictions and therefore improved estimations of attributable heat-related mortality^231^. Future urban research is likely to combine and harmonise data from various scales and sources, and leverage Spatial Data Science and AI-driven technologies to gain a more comprehensive understanding of urban dynamics, challenges and solutions.

### 4.3 What knowledge gaps necessitate further exploration?

A minority of studies included SE and demographic variables in analyses; however, observations from those that did confirm social determinants are an important avenue of future urban environmental health research. This would advance understanding of whether distinct urban form types can mitigate inequalities. Further, investigating inequalities within cities is particularly important in light of the limited knowledge of vulnerability drivers responsible for across city variation. These differences can be important; for example, differences in air pollution-attributable health burden are mostly due to differential levels of pollutants and can partly be explained by the pollutant chemical compositions^232^, whereas for other drivers, such as temperature, differences can be due to the level of vulnerability and resilience of the population^233^.

The paucity of demographic and SE data available at local-level was a commonly cited reason for not examining between population-group differences. This dearth of data both impedes the identification of health disparities and undermines the formulation of targeted and effective public health strategies for vulnerable populations. This is reflected in the literature from the limited evidence on gender-specific outcomes from urban adaptation intervention^234^. Females have been shown to experience multiple barriers to public transportation accessibility and thus this may influence female commuting choices and in turn exposure levels^235^. For HIAs, a methodological challenge central to the tendency of not stratifying estimates by gender and age is the lack of available sub-group ERFs. This reflects a gap in the underlying epidemiological evidence^236^. The lack of age-specific ERFs, particularly for populations under 20 years, may also be a by-product of the overemphasis on PM_2.5_ and O_3_ pollutants in the literature. PM_2.5_- and O_3_-realated mortality impacts generally focus on the over 25-year-old population; however, in recent years more research has emerged for NO_2_-related health outcomes in paediatric populations^123^,^195^.

### 4.4 Limitations of urban environmental health studies

The pathways covered in this review are not an exhaustive list and do not cover all pathways to health. Additional pathways that hold relevance include social exclusion^237^, community severance^237^, stress^237^, and proximity to blue space^238^. There was an evident paucity of research investigating health burden attributed to noise pollution. The only noise study analysed impacts from road traffic noise; however, aircraft, rail and construction noise also have considerable health impacts^239^,^240^. The household noise annoyance indicator may capture some of this exposure; however, the finest spatial resolution of NUTS3 restricts inferences for within city variability (Table A1). No studies incorporated climate change risk, which is a notable limitation for the HIAs that projected extreme heat and UHI.

The majority of studies applied regional-level estimates at city-level and assumed uniform distribution across cities, which discounts variability within and between cities. Commonly cited reasons for applying regional estimates were inconsistent data quality and availability at local-level and finer spatial resolutions^123,125,130^; however, this can introduce the risk of uncertainty in local impact predictions. Approaches to mitigate this included extrapolating metrics from geographies with greater data coverage^121,142^ or excluding geographies from analyses^46^. The latter pertains to the significant challenge of conducting HIAs in low- and middle-income countries^241^. Few studies investigated within-city variation^47,52,54,55,60^; the extent of which was also subject to data availability and quality^47^. Ensuring fairness in data exploration and identification of local inequities necessitates robust and comprehensive datasets with uniform data collection at local-level. Central to this is collaboration across sectors, levels of government, and for researchers and practitioners to leverage open-data platforms^205^.

Applicable to all HIAs was the uncertainty attributed to ERFs and RRs. There was high variation in ERF data sources, which points to the general uncertainty surrounding the selection of the most accurate ERFs to apply (Table 4). For the majority of HIAs, the same ERFs were applied to the general population, which assumes equivalent risk. The paucity of sub-group ERFs that capture susceptibility merits that recommendations cannot be made for susceptible subpopulations.

### 4.5 Strengths and caveats of review

This was a scoping and not a formal systematic review, and therefore, aimed to provide a holistic overview of evidence from large-scale urban studies, rather than assess all evidence concerning a single relationship (e.g., air pollution and birth weight). Inclusion of additional health outcomes (e.g., mental health) in search terms may have identified further large-scale urban studies of relevance. Investigation of the interplay between urban environments and both established and emerging infectious diseases was beyond the scope of this review; however, these pathways have high relevance to the complex urban health ecosystem. Changes to land use, demographic shift patterns, and globalisation infrastructures have been identified as pivotal factors that influence infectious disease incidence and outbreak^242^. The COVID-19 pandemic illustrates the crucial role of governments and policies in managing infectious disease outbreaks, and highlights the inevitable trade-offs and conflicts encountered in planning strategies^243^. Enhancing understanding of the interconnection between urban form and infectious diseases holds significant prominence in both research and governmental priorities for urban and transport planning. The scope of exposures included in this review aligned with those of the UBDPolicy project^45^; however, the caveat of additional pathways being excluded pertains to the broader challenge of prioritisation and resource constraints. Initiatives such as Urbanisation and Health Initiative^244^ led by the WHO, and the Urban Health Collaborative^245^ led by Drexel University, recognise the significance of investigating non-communicable and infectious diseases in tandem.

Strengths of this review include the expert consultation of relevant literature, which extended the scope of reviewed studies, and inclusion criterion of large-scale urban studies, which serves to increase the reliability and generalisability of results. Equally, this may have been a limitation as potential insights may have been missed from the 90-city inclusion criterion. Studies of fewer cities may have covered understudied regions and vulnerable populations. Not all geographical regions were covered (for example Australia and South Asia) and only English search terms were included in the literature search, exclusion of studies conducted in other languages may have contributed to the geographic distribution of studies and introduced bias in reported results. However, 22 studies were global in geographic coverage, this is considered a strength and may have mitigated potential exclusion bias. Further, PubMed was the sole electronic database articles were obtained from. This was due to PubMed’s comprehensive coverage of health and biomedical research. Finally, examination of urban policies and affiliated impacts was beyond the scope of this review.

## 5 Conclusion and Future Perspectives

This scoping review aimed to synthesise evidence from large-scale urban studies to provide a state-of-the-art overview of the relation between urban structures, transport, environmental exposures, and health. The complexity of the urban ecosystem was evidenced and emphasises the need for a multi-faceted approach for elucidating the intricate urban environmental health pathways. Researchers should prioritise exploring associations at multiple spatial scales and resolutions, both within and between population groups. Identifying local disparities in exposure, vulnerability, and adaptation will require enhanced local-level data, open-source indicators, and shared consensus of best research practices. Advances in techniques, temporal trend analysis, and urban health and sustainability indicators show promising developments. To fully harness the potential of cities as key drivers of sustainable and healthy living, robust evidence should spearhead this change. Only then can policies and interventions realise the impact they set out to achieve.

## Supplementary Material

Table A1

## Figures and Tables

**Table 1:** Summary of search terms and results for review.

Search terms			Theme	PubMed^a^	Included^b^	Total included^c^
			**Search 1**			
Urbanisation Urban typology Urban type Urban studies Urban environment Built environment Urban morphology Urban configuration Urban form Urban areas Cities Sprawl Urban planning Urban development Urban design Urban factors Urban features Urban characteristics Urban density Urban land use Urban land cover	Health Health impacts Health effects Health impact assessment Mortality Morbidity Disease		Urban form and health	2513	7	9
			**Search 2**			
	Health Health impacts Health effects Health impact assessment Mortality Morbidity Disease	Air pollution Particulate matter Nitrogen Dioxide PM2.5 NO2	Air pollution and health impacts	201	9	29
	Health Health impacts Health effects Health impact assessment Mortality Morbidity Disease	Urban heat island Temperature Heat	Temperature and health impacts	124	7	8
	Health Health impacts Health effects Health impact assessment Mortality Morbidity Disease	Green space Greenness Tree canopy Tree cover Park Urban green infrastructure Nature-based solutions Green infrastructure Green interventions Urban forests NDVI	Green space and health impacts	18	3	5
	Health Health impacts Health effects Health impact assessment Mortality Morbidity Disease Annoyance Sleep disturbance	Noise Road traffic noise Environmental noise	Noise and health impacts	16	1	1
	Health Health impacts Health effects Health impact assessment Mortality Morbidity Disease Injury Accidents Physical activity	Urban mobility Urban transport Road transport Urban travel Travel patterns	Transport and mobility	2	1	2
	**Search 3**					
		Indicator Indicators	Indicators	84	2	12

The same search terms relating to “urban form” were included in all searches.

a Values denote the total number of articles obtained from the respective search terms, for each search performed.

b Values denote the number of relevant articles included from PubMed search, following exclusion. Exclusion was based upon studies analysing < 90 cities, or not specifically assessing health impacts. The exclusion criteria did not apply to articles focused on indicators.

c Values denote the total number of included articles, by theme, after a supplementary search using included article reference lists and from expert consultation.

**Table 2:** Summary of 66 included studies, by theme, geographic scope, and number of cities analysed.

Theme	Theme subcategory	Environmental Exposures	Number of studies	Geographical regions covered (Number of studies)	No. of cities Mean / Median (range)
Urban form and health	-	-	9	Global (2) Africa (2) Latin America (4) United States (1)	1046 / 363 (110-5625)
	Urban environmental health	Air pollution	8	Global (4) China (1) Latin America (3)	312 / 346 (117-462)
		Temperature	6	Global (1) Europe (2) Latin America (2) United States (1)	447 / 500 (209-601)
		Green space	4	Africa (1) Europe (2) United States (1)	2118 / 496 (233-5625)
		Noise	-	-	-
Urban environmental health		Transport and mobility	2	Global (2)	997 (301-1692)
	Health Impact Assessment	Air pollution	21	Global (6) China (13) Europe (2)	2048 / 335 (95-13189)

		Temperature	2	Europe (2)	474 / 474 (93-854)
		Green space	1	Europe (1)	978
		Noise	1	Europe (1)	724
		Transport and mobility	-	-	-
Indicators	-	-	12	Global (9) Europe (3)	288 / 27 (14-1038)

**Table 3:** Summary of urban form, environment, and health studies that analysed at least 90 cities (cities analysed ranged from 110 – 5,625).

Theme	Reference	Location (number of cities)	Study design	City definition	City database	Health outcome	Health data source	Environmental Exposure	Exposure data source	Urban form metric	Data source	Statistical method^a^
**Urban form and health**	Prieto-Curiel et al., 2017^51^	Africa (1939)	Modelling	Continuously built-up area with <200m between two buildings and ≥10,000 inhabitants	Africapolis^75^	-	-	-	-	City size Market potential Urbanisation level Local dominance	Africapolis^76^	-
						**Main findings**						
						- Spatial clustering classified seven city groups that showed distinct urbanisation dynamics and regional interactions. - Spatial variables influenced urban growth rates, the emergence of urban agglomerations, and the clustering of cities.						
	Prieto-Curiel et al., 2017^52^	Africa (5625)	Modelling	Continuously built-up area with <200m between two buildings and ≥10,000 inhabitants	Africapolis^75^	-	-	-	-	Building height Street network metrics Terrain metrics	Google AI Africa Open Buildings dataset	BASE model^b^
						**Main findings**						
						- Through estimation of interbuilding distances and urban form metrics, the cumulative effects of increased number of buildings, increased building size and sprawl were assessed. - Estimated how increased urban commute times translates to required energy demand. - When a city population doubles, energy demand from transport was found to triple.						
	Bilal et al., 2021^50^	Latin America (363)	Ecological	Agglomerations of administrative units with ≥100,000 residents	SALURBAL study^77^	Cancer-related mortality CVD and other NCD-related mortality Unintentional injury-related mortality Violence-related mortality	Vital registration systems	-	-	City size City growth Population density Fragmentation Street connectivity Social environment index	SALURBAL study^77^	Nonparametric approach Three-level negative binomial multilevel model
						**Main findings**						
						- Life expectancy and unintentional and violent injuries and deaths varied across cities, with large within-country variation. - Causes of death from communicable, maternal, neonatal and nutritional, cancer, CVD and other NCDs varied substantially between countries. - Rate ratios for each cause of death were associated with 1 standard deviation increase in city-level factors. - Dense cities were found to have more violent deaths (relative to CVD and NCDs). - Less fragmented and more connected cities had more communicable, maternal and neonatal and nutritional causes of deaths (relative to CVD and NCDs).						
	Mullachery et al., 2022^56^	Latin America (363)	Cross-sectional	Agglomerations of administrative units with ≥100,000 residents	SALURBAL study^77^	Healthcare-amenable mortality	SALURBAL study^77^			City population Fragmentation Patch density Population growth	SALURBAL study^77^	Log regression model
						**Main findings**						
						- Urban population size and fragmentation were associated with amenable mortality. - Regardless of fragmentation, population size was associated with higher amenable mortality. - In small cities, higher urban fragmentation was associated with lower amenable mortality. In large cities, higher urban fragmentation was associated with higher amenable mortality. - Population growth and higher SES (city-level) was associated with lower amenable mortality.						
	Nguyen et al., 2019^55^	United States (500)	Cross-sectional	Categorised into tertiles	United States Census Bureau^78^	Obesity Diabetes Self-rated health Mental distress Physical distress Physical inactivity Teen births	BRFSS Survey Data^79^	-	-	Highway Rurality Grassland	Google Street View	Linear regression models
						**Main findings**						
						- At country level, greater presence of highways was related to lower chronic diseases and premature mortality. - Similar adverse associations observed at country level were observed at census tract level for neighbourhood areas of less urban development.						
	Ortigoza et al., 2021^49^	Latin America (286)	Cross-sectional	Agglomerations of administrative units with ≥100,000 residents	SALURBAL study^77^	Infant mortality rate	Vital registration systems	-	-	Population size Population growth rate Living conditions score Services provision score Mass transit availability	SALURBAL study^77^	Poisson multilevel model
						**Main findings**						
						- Greater population size was associated with higher IMR (p-value 0.0017). - 6% (3.7 – 8.3%) higher population growth, 14.1% greater living conditions (9.2 – 18.6%), 11.4% better service provision (6.4 – 16.1%) and 6.6% (3.9-9.2%) mass transit availability associated with lower IMR. - No association was found between educational attainment (population-level) and IMR.						
	Taubenböck et al., 2020^80^	Global (110)	Modelling	Morphological urban areas	United Nations^81^	-	-	Local Climate Zones	ESA^82^	-	-	-
						**Main findings**						
						- City types were classified into 7 types based on global diversity of spatial urban configurations. - The distinct city types largely aligned with common geographic-cultural spaces. - Certain clusters were more spatially complex (e.g., African-American or Asian-African clusters). - 21 of 22 European cities belonged to cluster 3: medium-sized cities of high structural variability, medium compact, mid-rise and medium share of open space. - Findings confirmed similar cultural, SE, demographic or political factors produce similar morphologic-spatial urban configurations.						
	Avila-Palencia et al., 2022^53^	Latin America (230)	Cross-sectional	Agglomerations of administrative units with ≥100,000 residents	SALURBAL study^77^	NCD-specific mortality Unintentional injury-specific mortality	Vital registration systems	NDVI PM_2.5_ NO_2_ Carbon footprint	SALURBAL study^77^	Fragmentation Urban isolation Shape of patches	SALURBAL study^77^	Linear regression models
						**Main findings**						
						- Higher city fragmentation was associated with higher odds of having HTN (1.11; 1.01-1.21). - Presence of mass transit in the city was associated with higher odds of having HTN (1.30; 1.09-1.54). - Higher sub-city intersection density was associated with higher odds of having HTN (1.09; 1.04-1.15). - Higher sub-city population density was associated with lower odds of having HTN (0.90; 0.85-0.94).						
**Air pollution and impacts**	Meng et al., 2021^63^	Global (398)	Ecological	-	MCC^83^	All-cause mortality CVD mortality Respiratory mortality	Local authorities	NO_2_	MCC^83^	-	-	Time series quasi-Poisson generalised linear regression model Multi-level meta-analytical approach
						**Main findings**						
						- On average, 10μg/m^3^ increase in NO_2_ concentration on lag 1 previous day was associated with all-cause mortality (0.46%: 0.36-0.57%), CVD-related mortality (0.37%: 0.22-0.51%) and respiratory-related mortality (0.47%: 0.21-0.72%). - Associations remained robust after adjusting for co-pollutants (PM_10_ < 10μg/m^3^ and PM_2.5_ < 2.5μg/m^3^, ozone, SO_2_ and CO).						
	Ye et al., 2021^64^	China (367)	Ecological	Boundaries defined in the Population Census	China Health Statistical Yearbook^84^	All-cause mortality	China Health Statistical Yearbook^76^	PM_2.5_ PM_10_ CO_2_ NO_2_ SO_2_ TSP	China’s National Urban Air Quality Real-time Publishing Platform^85^	-	-	Random Forests model
						**Main findings**						
						- Compared air quality during the COVID lockdown period in early 2020 with a business-as-usual scenario and found: 1239 (844 - 1578) PM_2.5_ related avoidable deaths; economic savings 1.22 billion USD. 2777 (1565 - 3995) PM_10_ related avoidable deaths; economic savings 2.60 billion USD. 1587 (98 - 3104) CO related avoidable deaths; economic savings 1.36 billion USD. 4711 (3649 - 5781) NO_2_ related avoidable deaths; economic savings 4.05 billion USD. 213 (116 - 314) O_3_ related avoidable deaths; economic savings 0.20 billion USD. 1088 (774 - 1421) SO_2_ related avoidable deaths; economic savings 0.95 billion USD.						
	Kephart et al., 2023^60^	Latin America (326)	Cross-sectional	Clusters of administrative units encompassing an urban built-up area ^a^	SALURBAL study^77^	-	-	NO_2_ NDVI	SALURBAL study^77^ US Geological Survey (MODIS MOD13Q1)^86^	Population density Intersection density GDP per capita Traffic congestion	SALURBAL study^77^ Kummu et al., 2017^87^ Delclòs-Alió et al., 2019^88^	Multilevel models
						**Main findings**						
						- 85% of the study population (almost 9 out of 10 residents) were exposed to ambient NO_2_ concentrations that exceeded current WHO guidelines. - Larger, denser, and more congested cities had higher NO_2_ concentrations. - Higher population density was independently associated with higher NO_2_ concentrations (city and neighbourhood levels). - Greenness was associated with lower NO_2_ at neighbourhood level (not city-level). - Found a positive association between educational attainment (neighbourhood level) and ambient NO_2_ concentrations.						
	Heydari et al., 2022^65^	Global (117)	Meta-analysis	-	-	COPD Diabetes IHD Lower respiratory disease Lung cancer Stroke	GBD 2017^89^	PM_2.5_	WHO^90^	-	-	Non-linear Integrated Exposure Response function
						**Main findings**						
						- Eliminating traffic emissions was estimated to achieve WHO 2021 recommended PM_2.5_ levels for 25 cities, that had low current PM_2.5_ concentrations. - For cities with up to 30 – 40μg/m^3^of PM2.5 concentrations, the benefits of preventable mortality showed an increasing trend. After this threshold large variations in preventable mortality were observed. - The percentage reduction in diabetes-related mortality decreased with increasing PM_2.5_ concentrations (an opposing trend to other outcomes under study). - The IER functions of PM_2.5_ showed reduced health benefits at higher concentrations. - The shape of IER functions had a significant effect on health benefits.						
	Gouveia et al., 2021^59^	Latin America (366)	Cross-sectional	Urban clusters with ≥100,000 inhabitants	Global Urban Footprint Dataset^91^	-	-	PM_2.5_ NDVI	Atmospheric Composition Analysis Group^92^ US Geological Survey (MODIS MOD13Q1)^86^	Population density Fragmentation Mass transit Infrastructure City size City growth Intersection density	SALURBAL study^77^	Linear mixed models
						**Main findings**						
						- 58% (∼172 million) of the study population lived in urban areas with air pollution levels that exceeded the 10μg/m^3^ annual WHO recommended level. - Larger cities, cities with higher GDP, higher motorisation rate and higher congestion had higher PM_2.5_ concentrations. - Cities with higher population density had lower levels of PM_2.5_. Inclusion of motorisation rate attenuated the association. - Higher intersection density was associated with higher PM_2.5_ at sub-city level. - More green space was associated with lower PM_2.5_ at sub-city level. - Adjusting for all exposures found higher city per capita GDP and higher sub-city intersection density remained associated with higher PM_2.5_ levels.						
	Rezaei & Millard-Ball 2023^62^	Global (462)	Cross-sectional	Urban Centres with ≥1,500 inhabitants per km^2^	GHSL^93^	-	-	PM_2.5_ NDVI	GHSL^93^ Landsat annual Top-of-Atmosphere (TOA) reflectance composite	Weighted population density Compactness Street connectivity 2016 GNI per capita	Global Human Settlement Layer^93^ OpenStreet Map Network^94^ World Bank^95^	Random Forest regression
						**Main findings**						
						- Correlations between urban form metrics were context specific, and therefore the impacts of urban form characteristics were not generalisable from one income group or geographic region to another. - No association was found between urban form metrics and transportation emissions per capita. - There was higher variation in emissions exposure between income groups. - Street connectivity had the strongest association with reduced PM_2.5_ emissions from the transportation sector. - In Europe, street connectivity was correlated with higher population density.						
	Avila-Palencia et al., 2022^69^	Latin America (208)	Ecological	Agglomerations of administrative units with ≥100,000 residents	SALURBAL study^77^	NCD-specific mortality Unintentional injury-related mortality HTN Diabetes Obesity	Vital registration systems National surveys WHO 2016^96^	PM_2.5_ NO_2_ Carbon footprint NDVI	Atmospheric Composition Analysis Group^92^ Moran at al., 2018^97^	Fragmentation Isolation Shape of urban patches	SALURBAL study^98^	Spearman correlations Linear regression models
						**Main findings**						
						- Types of urban form were related to positive or negative health and environmental co-benefits. - 27% (56 cities) found to have positive co-benefits, and were generally small to medium sized with high population densities. - 44% (91 cities) found to have negative co-benefits. - Urban form type with the most co-benefits had low fragmentation, high isolation, and more compact development. - Urban form types that were least likely to be in the positive co-benefit class were higher fragmentation and complex shapes.						
**Temperature and impacts**	Kephart et al., 2022^67^	Latin America (326)	Ecological	Agglomerations of administrative units with ≥100,000 residents	SALURBAL project^77^	All-cause mortality CVD-related mortality Respiratory disease-related mortality Respiratory infection-related mortality	Vital registration systems	Ambient air temperature	ERA5-Land^99^	-	-	Distributed lag nonlinear conditional Poisson model Random effects meta-regression model
						**Main findings**						
						- Overall, higher proportion of deaths were attributable to ambient cold compared to ambient heat. - Risks were strongest among older adults and for CV- and respiratory-related deaths. - RR 1.057 (1.046 – 1.067) per 1°C higher temperature during extreme heat. - RR 1.034 (1.028 – 1.040) per 1°C lower temperature during extreme cold. - For heat-related deaths, 0.67% (0.58 – 0.74) excess death fraction of total deaths. - For cold-related deaths, 5.09% (4.64 – 5.47) excess death fraction of total deaths.						
	Wang et al., 2016^100^	United States (209)	Ecological	-	-	Mortality	National Centre for Health Statistics	Cold waves^c^	CMIP Phase 5^101^	-	-	Over-dispersed Poisson regression
						**Main findings**						
						- Cold waves were associated with a small increase in risk of mortality. - Lingering effects of cold waves were larger than the cold waves themselves. - Risk increased with duration and intensity of cold waves, however decreased with mean winter temperature. - Associations varied substantially across climatic regions.						
	Krummenauer et al., 2019^61^	Europe (599)	Ecological	≥1,500 inhabitants per km^2^	Gridded population of the world^102^	Life expectancy Health expenditure	WBOD^95^ World Income Inequality Database^103^ MDGLR^104^	Minimum mortality temperature	Global Summary of the Day^105^	Topography Population density GDP per capita GINI coefficient Improved water source	CIESIN^102^	Non-linear sigmoid model
						**Main findings**						
						- MMT was found to be influenced by topography and SE factors. - There was lower MMTs in cities with higher altitudes. - There was a positive association between higher SE indicators with MMT, suggesting higher SES increases an urban population’s adaptive capacity to heat. - Other climatic, topographic, demographic and SE factors were not significant predictors of MMT.						
	Alahmad et al., 2023^66^	Global (567)	Ecological	-	MCC^83^	CVD-specific mortality data	MCC^83^	Ambient temperature	MCC^83^	-	-	Case-crossover models Mixed-effects meta-analytic framework
						**Main findings**						
						- Extreme heat and cold were associated with a higher risk of dying from any CVD-cause, IHD, stroke, and HF compared to MMT. - Excess CVD deaths from sustained extreme cold were larger than those from extreme heat. - For every 1000 HF deaths, hot days accounted for 2.6 (2.4-2.8) deaths and cold days accounted for 12.8 (12.2 – 13.1) deaths. - For every 1000 CVD deaths, cold days (below 2.5^th^ percentile) accounted for 9.1 (8.9-9.2) and hot days (above 97.5^th^ percentile) accounted for 2.2 (2.1 – 2.3).						
	Bakhtsiyarava et al., 2023^58^	Latin America (325)	Ecological	Agglomerations of administrative units with ≥100,000 residents	SALURBAL study^77^	All-cause mortality CVD-specific mortality	Vital registration systems SALURBAL study^77^	Temperature	ERA5-Land^99^	-	-	Distributed lag nonlinear conditional Poisson model Random effects meta-regression model
						**Main findings**						
						- Greater excess mortality was associated with cold temperatures (below MMT): 5.09% (4.64 – 5.47) compared to excess mortality associated with heat (temperatures above MMT): 0.67% (0.58 – 0.74%). - There was limited effect modification of demographic and SE characteristics (city-level) of cold-related mortality. - GINI index of income inequality was the only modifier to show a statistically significant association with all-age, cold-related mortality (3.45 [CI 0.33, 6.56] percentage-points higher compared to cities with a low GINI index). - Higher levels of poverty was associated with lower heat-related mortality: cities in the top tertile of population density had heat EDF 0.70 [CI 1.16, -0.25] percentage-points lower than cities in the bottom tertile. - Higher income inequality was associated with lower heat-related mortality: cities in the top tertile of the GINI index had heat EDF 1.16 [CI 1.90, -0.43] percentage-points lower than cities with the smallest GINI index.						
	Zhou et al., 2017^68^	Europe (5000)	Ecological	Urban agglomerations	CORINE land cover^106^	-	-	Surface UHI intensity	CMIP Phase 5^101^	City size Urban fractality Urban anisometry	CORINE morphological zones^107^	Multi-linear regression model
						**Main findings**						
						- Larger and more compact cities (high urban fractality) with less sprawl (small anisometry) had the strongest UHI intensities. - City size had the strongest influence on UHI, followed by fractality. - There was a complex interplay between urban form factors and UHI.						
**Green space**	Marando et al., 2022^72^	Europe (601)	Modelling	Functional Urban Areas	GHSL^93^	-	-	Land surface temperature	Google Earth Engine^108^	Cooling index^d^	Copernicus^109^ MODIS^86^	Bivariate linear regression model Univariate model
						**Main findings**						
						- Tree cover of at least 16% was required to achieve a reduction of 1°C in urban temperatures. - 32% of European FUAs had tree cover below 16%. - The impact of trees on reducing UHI is dependent on the extent of green areas and amount of transpiration inside a city. - In almost 40% of the countries under study, more than half of the resident population do not benefit from the microclimate regulation provided by urban tree coverage.						
	Browning et al., 2018^57^	United States (496)	Cross-sectional	-	500 Cities project^110^	Obesity Mental health	500 Cities project^110^	NDVI Tree cover	MODIS^111^ Multi-Resolution Land Characteristics Consortium^112^			Spatial moving average models
						**Main findings**						
						- Greener cities had less obesity and better mental health outcomes. - No evidence that tree cover was more strongly linked to positive health outcomes compared to greenness. - Cities with lower median household income had greater benefits from green space compared to wealthier cities. - Sprawl did not have a moderating effect on the greenspace-health link. - Regardless of a city’s population density, tree cover was linked to better obesity outcomes and overall greenness was linked to better mental health outcomes.						
	Anderson et al., 2022^10^	Africa (5625)	Modelling	Continuously built-up area with <200m between two buildings and ≥10,000 inhabitants	Africapolis^75^	-	-	Urban green space fraction Proximity to green space PM_2.5_	WorldClim^113^ GHSL^114^	Urban form metrics^e^	European Space Agency’s World Cover Map^115^	Linear econometric models
						**Main findings**						
						- None of the cities under study met the WHO 2005 recommended air quality levels. - If cities had at least 25% green space cover PM_2.5_ levels could reach moderately safe levels. - The benefits of green space availability were not the same as proximity to green space. Recommendations included varied-sized patches of green throughout the city.						
	Olsen et al., 2019^70^	Europe (233)	Cross-sectional	Large Urban Zones of ≥100,000 inhabitants	Urban Atlas 2018^116^	All-cause mortality (SMR)	Richardson et al., 2017^117^	-	-	Land cover uses^f^	See supplementary^70^	Linear regression models
						**Main findings**						
						- No evidence that the distribution of mixed land use was related to mortality rates. - The proportion of specific land use within a city was related to SMR. - Higher proportion of natural spaces, and less dense or non-residential land use was associated with lower mortality. - Relatively ‘wild’ green spaces (e.g., forest, wetlands, semi-natural areas) were associated with lower SMRs; this association was observed across sexes. - Dense housing was related to higher SMR, and was most prominently seen in Western European cities.						
**Transport and mobility**	Thompson et al., 2020^74^	Global (1692)	Cross-sectional	1) Minimum radius of 1.5km 2) Selected images of 400m^2^	United Nations^73^ Google Static Maps	Road traffic injuries (DALYs, YLLs, YLDs)	GBD 2016^110^	Fossil fuel emissions	FFDAS^111^			2 × 3 multivariate analysis of variance
						**Main findings**						
						- Identified nine global city types. - Urban design was strongly associated with the burden of road traffic injury. - Burden of road traffic injury was estimated to be two times higher for the poorest performing city type compared to the best performing city type. - Poorest performing city types included: cul-de-sacs, irregular, sparse and large block. - Best performing city type was high transit. - Estimated 9.6 million DALYs annually were attributable to suboptimal urban design.						
	Bassolas et al., 2019^73^	Global (301)	Ecological	Metropolitan areas	U.S. Census	Stroke (incidence) Stroke-related mortality Transport-related mortality	CDC^118^ US Department of Transportation^119^			Trip flow data	Mobility Map project^120^	Multivariate analysis
						**Main findings**						
						- Cities with larger mobility hierarchy showed more population mixing, extensive use of public transportation, higher levels of walkability, lower pollutant emissions per capita and better health indicators. - Asian and African cities were amongst the most hierarchal, followed by cities in Europe, America and Oceania. - Transportation in less hierarchal cities was dominated by private car use. - Important predictors of transportation included: spatial constraints, geographic limitations and land use.						

Abbreviations: Cardiovascular disease (CVD); Non-communicable disease (NCD); Social economic status (SES); Behavioural Risk Factor Surveillance System (BRFSS); Infant mortality rate (IMR); Normalised differential vegetation index (NDVI); Multi-City Multi-Country (MCC); Terra Moderate Resolution Imaging Spectroradiometer (MODIS) Vegetation Indices (MOD13Q1); Chronic obstructive pulmonary disorder (COPD); Ischaemic heart disease (IHD); Global Human Settlement Layer (GHSL); Hypertension (HTN); Coupled Model Intercomparison Project Phase 5 (CMIP5); World Bank Open Data (WBOD); Millennium Development Goals Lebanon Report (MDGLR); Centre for International Earth Science Information Network (CIESIN); Urban Heat Island (UHI); Standardised mortality rate (SMR); Disability-adjusted life years (DALYs); Years of life lost (YLLs); Years lived with disability (YLDs); Fossil Fuel Data Assimilation System (FFDAS).

a Statistical method for estimation of association between urban form, exposures, and health.

b BASE model: mean distance between buildings is a functional relation to the number of Buildings and their average Area and the Sprawl and the Elongation of its spatial arrangement. Allows relation of city morphology to distance indicators (e.g., sprawl, elongation, and polycentricity) and the energy demand from transport.

c Cold waves defined as two, three, or at least four consecutive days with daily temperature lower than the 5th percentile of temperatures recorded in each city.

d Variables included in cooling index: tree cover density, water evaporation from tree canopies, vaporisation of intercepted rainfall from vegetation.

e Urban form metrics include sprawl, city elongation, built-up intensity, intersection density, average node degree, city centre building density, types of green cover, total footprint centre 1km, is pyramid, urban green space fraction.

f Land covers/uses include agriculture, semi-natural areas, wetlands, green urban areas, industrial, commercial, public, military, discontinuous low density urban fabric, residential, isolated structures.

**Table 4:** Summary of health impact assessments that analysed at least 90 cities (cities analysed ranged from 93 – 13,189).

Reference	Location (number of cities)	City definition	City population database	Outcome	Outcome data source ^a^	Temporal resolution	Environmental exposure (Resolution Scale) ^b^	Environmental exposure data source	Relative Risk	ERF data Source ^c^	Models to estimate exposure	Counterfactual Scenario
Khomenko et al., 2021^5^	Europe (1016)	Local administrative boundaries, with ≥50,000 inhabitants^93^	Urban Audit^143^	Natural cause mortality (rate per 100 000 and YLL)	Eurostat^144^ (City-level)	2015	(100m^2^)	ELAPSE^145^	PM_2.5_-1.07 (1.04-1.09) per 10μg/m^3^ increase NO_2_-1.02 (0.99-1.06) per 10μg/m^3^ increase	WHO 2014^146^ Atkinson et al., 2018^147^	LUR model (100m^2^) Ensemble model (10km^2^) Global LUR model (100m^2^)	PM_2.5_-10μg/m^3^ NO_2_- 40μg/m^3^
Khomenko et al., 2023^124^	Europe (857)	Local administrative boundaries, with ≥50,000 inhabitants^93^	Urban Audit^143^	Natural cause mortality	Eurostat^139^ (City-level)	2015	PM_2.5_ NO_2_ (0.1°×0.05° /∼6km^2^)	Copernicus Atmosphere Monitoring Service regional inventory^148^	PM_2.5_-1.08 (1.06-1.09) per 10μg/m^3^ increase NO_2_-1.02 (1.01-1.04) per 10μg/m^3^ increase	Chen et al., 2020^149^ Huangfu & Atkinson 2020^150^	SHERPA tool^151^ EMEP MSC-W chemical transport model^152,153^	Pollutant concentrations related to each emission source eliminated
Anenberg et al., 2019^126^	Global (250)	Population census tables and corresponding geographic boundaries	CIESIN^102^	All-cause mortality IHD Stroke COPD Lung cancer Lower respiratory infections Diabetes	GHDx^148^ (0.1°×0.1° grid cell level)	2010 and 2015	PM_2.5_ Ozone (0.1°×0.1° /∼10km^2^)	ECLIPSE^154,155^	See references^156,89^	Shaddick et al., 2018^156^ GBD 2017^89^	GEOS-Chem global chemical transport model (2° x 2.5°)	PM_2.5_-2.4–5.9μg/m^3^ Ozone- 32.4 ppb (∼63.5μg/m^3^)
Zhang et al., 2022^140^	China (331)	Defined by the Population Census	China Health Statistical Yearbook^157^	Premature mortality CVD mortality Respiratory mortality	China Health Statistical Yearbook^157^	2015-2020	PM_2.5_ Ozone	China National Environmental Monitoring Centre^116^	ERF reported^140^	Kan et al., 2002^158^	Univariate linear regression model	PM_2.5_- 10μg/m^3^ Ozone- 26.7 ppb (∼54μg/m^3^)
Guan et al., 2021^139^	China (338)	Defined by the Population Census	National Bureau of Statistics of China^159^	All-cause mortality (DALY) Respiratory disease (DALY)	GBD Study 2016^160^ (Provincial level)	2015-2020	PM_2.5_ Ozone	China National Environmental Monitoring Centre^135^	All-cause ozone – 1.01 per 10μg/m^3^ increase Respiratory disease ozone – 1.02 per 10μg/m^3^ increase	Burnett et al., 2014^161^ Maji et al., 2018^138^ Wang et al., 2021^162^	-	PM_2.5_ – 10, 15, 25, 35μg/m^3^ Ozone– 100, 160μg/m^3^ (∼196, 313.6 ppb)
Guan et al., 2021b^125^	China (101)	City seasonal population	Baidu population migration index^163^	CVD (DALYs) Respiratory disease (DALYs)	GBD Study 2017^164^ (Provincial level)	Fourteen seasons from 2017, 2018, 2019 and first half of 2020	PM_2.5_ Ozone	Ministry of Environmental Protection^165^	See Table 1 of Appendix^125^	-	-	PM_2.5_- 25μg/m^3^ Ozone- 100μg/m^3^ (∼196 ppb)
Guan et al., 2022^166^	China (335)	Defined by the Population Census	National Bureau of Statistics of China^159^	All-cause (DALY) CVD (DALY) Respiratory disease (DALY)	GBD Study 2017^164^ (Provincial level)	2021	PM_2.5_ Ozone	China National Environmental Monitoring Centre^135^	-	Orellano et al., 2020^167^	-	PM_2.5_- 15μg/m^3^ Ozone- 70μg/m^3^ (∼137.2 ppb)
Anenberg et al., 2019^131^	Global (250)	≥1,500 inhabitants per km^2^	CIESIN^102^	Mortality	GBD 2016^89^	2016	PM_2.5_ ((∼0·0083°)^2^ /1km^2^) CO_2_ (1km^2^)	Shaddick et al., 2018^156^ Oda & Maksyutov, 2011^168^	Age-specific RR^c^	Cohen et al., 2017^169^	Chemical transport model (Calibrated to 6003 measurements for 117 countries)	2.4–5.9μg/m^3^
Maji et al., 2017^132^	China (190)	Defined by the Population Census	National Bureau of Statistics of China^170,171^	All-cause mortality 5 causes premature mortality 18 causes morbidity	GBD Study 2010^160^ (Provincial level)	2014-2015	PM_2.5_ PM_10_	GBD 2010^172^	See table 1^133^	GBD 2010^160^	-	PM_2.5_– 20μg/m^3^ PM_10_ – 5.8μg/m^3^
Maji et al., 2018^138^	China (338)	Defined by the Population Census	National Bureau of Statistics of China^170^	Stroke IHD COPD Lung cancer Cause-related hospital admission	GBD Study 2016^160^ (Provincial level)	2016	PM_2.5_	China National Environmental Monitoring Centre^135^	-	-	-	PM_2.5_ – 5.9μg/m^3^
Guan et al., 2019^173^	China (328)	Defined by the Population Census	National Bureau of Statistics of China^170^	CVD mortality Respiratory disease mortality Lung cancer mortality	Zhou et al., 2016^174^ (Provincial level)	2015-2017	PM_2.5_	China National Environmental Monitoring Centre^135^	-	-	-	PM_2.5_ - 10μg/m^3^
Diao et al., 2020^127^	China (338)	Defined by the Population Census^170^	China Health Statistical Yearbook^157^	All-cause mortality Respiratory mortality CVD hospitalisation Chronic bronchitis hospitalisation Asthma diagnosis Acute bronchitis diagnosis	-	2015	PM_2.5_	LandScan^175^	All-cause mortality PM_2.5_- 1.019 (1.003-1.081) per 10μg/m^3^ increase See Table 1 for full list^127^	Wang et al., 2017^176^	-	PM_2.5_- 10μg/m^3^
Han et al., 2022^128^	China (296)	Population census tables and corresponding geographic boundaries	CIESIN^102^	All-cause mortality	China Health Statistical Yearbook^157^ (City-level)	2015-2019	PM_2.5_ (0.1°×0.1° /∼10km^2^)	Satellite sources^177^ Emission-inventories^178^ Model simulation^179^ Ground-based sources^180^	All-cause mortality PM_2.5_-1.055 (1.022–1.088) ^139^ per 10μg/m^3^ increase	Zhang 2021^181^	Artificial intelligence combined data from satellite-, emission inventories-, model simulation- and ground-based sources.	PM_2.5_ - 5μg/m^3^
Southerland et al., 2022^129^	Global (13,160)	Defined by Global Human Settlement Model grid^182^	European Commission’s Joint Research Centre^183^	Attributable cause-specific mortality of: Ischaemic heart disease Intracerebral haemorrhagic stroke Lower-respiratory infections Lung cancer Type 2 diabetes COPD	GBD 2019^26^ (National level)	2000-2019	PM_2.5_ ((∼0·0083°)^2^ /1km^2^)	PM_2.5_ concentration database^184^	Produced RR estimates for 385 integer exposure levels ranging from 0-2500 μg/m^3^	Zheng et al., 2021^185^	Integrated data from satellite-retrieved aerosol optical depth, chemical transport modelling, and ground monitor data.	PM_2.5_ - 2.4-5.9μg/m^3^
Zhang et al., 2008^130^	China (111)	Defined by the Population Census	China Health Statistical Yearbook	All-cause mortality CVD hospitalisation Chronic bronchitis Acute bronchitis Respiratory hospitalisation Asthma attack Outpatient visits (internal medicine) Outpatient visits (paediatric)	China Health Statistical Yearbook^157^ (Provincial level)	2004	PM_10_	SEPAC^186^	ERF reported^130^	-	-	PM_10_ - 40μg/m^3^
Malashock et al., 2022^133^	Global (12,946)	Population of ≥0.05 million and ≥ 1500 inhabitants per km^2^, or built up area of at least 50% and town population between 20000-50000^183^	European Commission’s Joint Research Centre^183^	Attributable cause-specific mortality	GBD 2019^26^ (National level)	2000-2019	Ozone ((∼0·0083°)^2^ /1km^2^)	OSDMA8^187^	Respiratory mortality- 1.06 per 10 ppb ozone	Turner et al., 2016^188^	-	Ozone- 32.4 ppb^188^ (∼63.5μg/m^3^)
Guan et al., 2022^189^	China (338)	Defined by the Population Census	China Health Statistical Yearbook	All-cause mortality Respiratory mortality COPD mortality	GBD Study 2017^164^ (Provincial level)	2015-2020	Ozone NO_2_ (0.25°×0.25°)	China National Environmental Monitoring Centre^135^	-	Anenberg et al., 2018^190^ Huangfu and Atkinson 2020^150^	-	WHO 2021 guidelines^191^
Maji et al., 2019^192^	China (338)	Defined by the Population Census	China Health Statistical Yearbook^157^	CVD mortality Respiratory mortality	GBD Study 2016^160^ (Provincial level)	2016	Ozone	China National Environmental Monitoring Centre^135^	Respiratory mortality- 1.04 (1.013 - 1.067) per 20mg/m^3^ increase CV mortality- 1.01 (1 - 1.2) per 20mg/m^3^increase	Jerrett et al., 2009^193^	-	Ozone- 75.2μg/m^3^ (∼38.34 ppb)
Mead et al., 2006^134^	China (95)	Defined by the Population Census	China Environmental Yearbook	Non-accident mortality	Author derived (City-level)	2001	NO_2_ SO_2_ TSP	China Environmental Yearbook	NO^2^- 1.012 and 1.008 SO_2_- 1.0188 TSP- 1.013	-	-	NO_2_-80 and 40μg/m^3^ SO_2_- 60 and 50μg/m^3^ TSP- 200 and 90μg/m^3^
Anenberg et al., 2022^123^	Global (13,189)	Defined by Global Human Settlement Model grid	European Commission’s Joint Research Centre^183^	Paediatric asthma incidence	GBD 2019 study^26^ (National level)	1990-2019	NO_2_ ((∼0·0083°)^2^ /1km^2^)	Adjusted existing model (Larkin et al., 2017^194^)	1.26 (1.1-1.37) per 10 ppb annual average increase	Achakulwisut et al., 2019^195^	LUR model (100m^2^)	NO_2_ - < 2 ppb (∼3.78μg/m^3^)
Song et al., 2023^142^	Global (13,189)	Defined by Global Human Settlement Model grid	European Commission’s Joint Research Centre^183^	All-cause mortality	GBD 2019 study^26^ (City-level)	2019	NO_2_ (1km^2^)	Dataset from Anenberg et al., 2022^123^	1.047 (1.023-1.072) per 10 ppb increase	Stieb et al., 2021^196^	LUR model^123^	10μg/m^3^ (∼5.32 ppb)
Barboza et al., 2021^47^	Europe (978)	Local administrative boundaries, with ≥50,000 inhabitants^85^	Urban Audit^137^	Natural-cause mortality (rate per 100 000 and YLL)	Eurostat^192^ (City-level)	2015	NDVI %GA (250m^2^)	US Geological Survey (MODIS MOD13Q1)^78^ European Urban Atlas^129^	%GA–0.99 (0.98-1.01) for every 10% increase in GA NDVI–0.96 (0.94-0.97) for every 0.1 unit increase in green exposure	Gascon et al., 2016^193^ Rojas-Rueda et al., 2019^194^	-	%GA– 25% GA within 300m of residence Target NDVI estimated per city^40^
Iungman et al., 2023^39^	Europe (93)	Local administrative boundaries, with ≥50,000 inhabitants^93^	Urban Audit^143^	All-cause mortality (rate per 100 000 and YLL)	Eurostat^197^ (City-level)	2015	Heat (UHI) (100m^2^) Tree cover density (250m^2^)	Copernicus Urban Climate dataset^198^ Copernicus tree coverage^199^	City and age-specific ERFs; supplementary^46^	Masselot et al., 2023^121^	-	Day-time UHI-0.6ºC Night-time UHI- 1.9ºC Tree coverage: 25%, 30%, 40%
Masselot et al., 2023^121^	Europe (854)	Local administrative boundaries, with ≥50,000 inhabitants^93^	Urban Audit^143^	All-cause mortality Non-accidental causes of mortality	Eurostat^144^ MCC Collaborative Research Network^83^ (City-level)	2000-2020^d^	Extreme heat Extreme cold (9km^2^)	ERA5-Land dataset^99^	City and age-specific ERFs; see supplementary^121^	Masselot et al., 2023^121^	-	-
Khomenko et al., 2022^6^	Europe (724)	Local administrative boundaries, with ≥50,000 inhabitants^93^	Urban Audit^143^	High noise annoyance IHD (rate per 100 000 and YLL)	Guski et al., 2017^200^ Eurostat^144^ (City-level)	2015	Road traffic noise (250m)	Environmental Noise Directive^201^	IHD-1.05 (0.97-1.13) per 10dB increase	Van Kempen et al., 2018^202^	Country-specific prediction models (250m^2^) using ordered logistic regression for aggregated data.	53dB

Abbreviations: Years of life lost (YLL); Effects of low-level air pollution: a study in Europe (ELAPSE); Land Use Regression (LUR); Screening for High Emission Reduction Potentials for Air Quality (SHERPA); European Monitoring and Evaluation Programme for Transboundary Long-Range Transported Air Pollutants Meteorological Synthesizing Centre-West (EMEP MSC-W); Ischaemic heart disease (IHD); Chronic obstructive pulmonary disorder (COPD); Global Health Data Exchange (GHDx); Cardiovascular disease (CVD); Disability-adjusted life years (DALYs); Global Burden of Disease Study (GBD); State Environmental Protection Administration of China (SEPAC); Total suspended particles (TSP); Normalised differential vegetation index (NDVI); Terra Moderate Resolution Imaging Spectroradiometer (MODIS) Vegetation Indices (MOD13Q1); Urban heat island (UHI).

a Spatial scale denotes the finest level of analysed health data. Resolution scale denotes the grid-cell level the exposures were estimated at, when reported.

b ERF source used to calculate relative risk.

c Age-specific RR calculated for each grid cell PM_2.5_ concentration not reported, available from the authors upon request.

d Average taken from 20-year time series and therefore was not a trend analysis.
