# Supplementary material for: Exploring the nexus of urban form, transport, environment and health in large-scale urban studies: a state-of-the-art scoping review
Source: Environ Res. Author manuscript; Available in PMC 2025 Jun 7. (PMC7617738; doi:10.1016/j.envres.2024.119324)
Supplement: Table A1 [file EMS205650-supplement-Table_A1.docx]

**Appendix**

**Table A1** Themes and indicators identified in this review.

| **Theme** | **Indicator** | **Description** | **Methods** | **Geographical coverage** | **Spatial resolution** | **Data Sources** |
| --- | --- | --- | --- | --- | --- | --- |
| **Urban form** | Recreational space per capita^211^ | The hectares of recreational space (open space for public use)  per 1,000 people. | Recreational space data retrieved from OSM.  OSM tags are employed to retrieve polygons that delineate areas of parks, nature reserves, commons, playgrounds, pitches, tracks, protected areas and national parks.  Population data retrieved from WorldPop.  The total recreational area within a jurisdictional boundary was divided by the population within the boundary per 1000 individuals. | Global | - | WorldPop^246^  OpenStreetMap^247^ |
|  | Urban open space for public use^211^ | The percentage of built-up area that is open space for public use. | Recreational space data retrieved from OSM.  OSM tags are employed to retrieve polygons that delineate areas of parks, nature reserves, commons, playgrounds, pitches, tracks, protected areas and national parks.  Definition of urban open or non–open space for each 10m pixel of built land derived using the built-up from ESA.  The ratio of masked pixels representing open space to the total count of masked pixels was used to calculate the percentage of built area designated to open space. | Global | 10m | OpenStreetMap^247^  ESA WorldCover^248^  Zanaga et al., 2021^115^ |
|  | Proximity to public open space^211^ | The percentage of the population within walking distance (400m) of public open space. | Utilised the gridded population (100m).  Retrieved open space polygons from OSM buffered to 400m to derive recreation catchment areas.  The population residing within the recreation catchment areas was determined and converted into a percentage by dividing that value by the total population of the area of interest. | Global | 400m^2^ | WorldPop^246^  OpenStreetMap^247^ |
|  | Proximity to tree cover^211^ | The percentage of the population with an average tree cover  of greater than 10 percent within walking distance (400 m)  of their homes. | Utilised 10m resolution tree cover and the gridded population (100m).  A neighbourhood reduction technique utilising a circular kernel with radius 400m was employed to the tree cover layer to determine the average percentage of tree cover within a 400m radius of each 10m pixel within the area of interest.  The result is subsequently applied to filter the population layer, restricted to include 100m population pixels with an average tree cover of more than 10 percent within a 400m radius.  The population within the 100m masked population layer is calculated and then converted to a percentage by dividing this figure by the total population of the area of interest. | Global | 400m^2^ | Mosaic Landscapes data set^249^  WorldPop^246^ |
|  | Distance to local amenities^205^ | Percentage of population living within 500m of a fresh food market, a convenience store, and public transport. | Developed indicators for pedestrian network distance accessibility within a 500m radius, assessed for hexagonal grid cells and adjusted based on population percentage estimates. | Global  (25 cities) | 500m | Global Human Settlement Layer^93^  Custom boundaries (see Appendix^205^)  OpenStreetMap^250^ |
| **Air pollution** | High pollution days^211^ | Annual number of days that air pollutants were above WHO air quality guidelines in 2020. | The extracted data combines satellite monitoring of pollutant concentrations with atmospheric modelling to estimate concentrations in close proximity to the Earth’s surface.  Reported the number of days in 2020 for each city that had near-surface concentrations of air pollutants that  Exceeded WHO’s guidelines for outdoor air pollutants^251^. | Global | 80km | CAMS Global  Reanalysis EAC4^252^ |
|  | Fine particulate matter exposure^211^ | Annual mean PM_2.5_ concentration  as a percentage of WHO’s air quality guideline for  annual exposure. | Extracted data combines models of atmospheric mixing and chemistry with imagery analysis (from the Moderate Resolution Imaging Spectroradiometer and Sea-viewing Wide Field-of-view Sensor satellite instruments from NASA) to generate estimates of PM_2.5_ concentrations near the earth’s surface, based on annual average concentrations for 2020.  Each district’s 2020 average PM_2.5_ concentration reported as  a percentage of WHO’s air quality guideline for annual exposure of 5μg/m^3^.  The annual average is calculated over the  area of the district. For example an average concentration of 15μg/m^3^ would be reported as 300 percent of the WHO guideline. | Global | 0.01° (~1.1km) | Atmospheric Composition Analysis Group^95^ |
|  | Long-term exposure to PM_10_^209^ | Number of days particulate matter PM_10_ concentrations exceed 50 µg/m³. | Calculated the sum of total days that PM_10_ concentrations exceeded 50µg/m^3^ for 2016. | Europe  (28 cities) | NUTS3 | Urban Audit^143^ |
|  | Annual NO_2_ exposure^209^ | Annual average concentration of NO_2_ (µg/m³) | Calculated the average annual concentration of NO_2_ for 2016. | Europe  (28 cities) | NUTS3 | Urban Audit^143^ |
| **Temperature** | Built land without tree cover^211^ | The percentage of built land without tree cover. | Tree cover with resolution of 10m applied. Built-up land data was obtained from ESA WorldCover and used to mask the tree cover layer.  Counted the number of built area pixels that also had tree cover, and the total number of pixels with built areas. These two values were divided to determine the percentage of built land covered by trees.  The percentage of tree cover was inverted to calculate the percentage of built-up land that lacked tree cover. | Global | 10m | Mosaic Landscapes data set^249^  ESA WorldCover 2020^115^ |
|  | Extreme heat hazard^211^ | The anticipated extreme heat event hazard (measured as the number of days above  35°C in 2050) and the trend (indicated by the percentage change in the number of days exceeding 35°C between 2020 and 2050). | Calculated the anticipated number of days with maximum  near-surface air temperatures exceeding 35°C, for 2020 and 2050. Subsequently subtracted the 2020 estimate from the 2050 and divided this difference by the 2020 estimate and multiplied the result by 100.  The resultant value is calculated from a probability distribution model. | Global | 0.25° pixel containing the city centroid | ERA5 global reanalysis^253^  NEX-GDDP ensemble climate projections^254^ |
|  | Land surface temperature^211^ | Percentage of built-up land with a high LST during the hot season (greater than or equal to 3°C above mean for built-up land). | LST calculated or each pixel in the area of interest  using methods described elsewhere^255^ and Landsat imagery.  Average LST is calculated from a compilation of Landsat images that are cloud-masked. Images span from 2013 to 2022 and are selected for each year from the month with the highest temperature recorded, as determined by the ERA5 daily aggregates^253^.  Average pixel LST were retrieved for built-up land cover areas, classified by the ESA WorldCover.  Areas where the temperature exceeded the area average by 3°C or more were excluded to determine the proportion of build-up areas with elevated LST. | Global | 30m | Google Earth Engine^255^  ESA WorldCover 2020^115^ |
|  | Surface reflectivity^211^ | The percentage of built-up land with low surface reflectivity. | Used pixel-wise albedo values derived from Sentinel-2 using the algorithms defined elsewhere^256^.  Annual mean albedo was calculated using cloud-free pixels from 2021. Values for built-up land cover were obtained by applying the built-up class from the ESA WorldCover dataset as a masking tool.  Pixels with values lower than 0.2 were excluded to determine the proportion of built-up area with reduced surface reflectivity. | Global | 10m | Google Earth Engine^255^  ESA WorldCover 2020^115^ |
| **Green space** | Open or green space^202^ | Percentage of population living within 500m of a public open space | For data obtained from OSM, followed tagging guidelines and collaborator feedback to classify open or green spaces.  Determined the percentage of population residing within 500m of a public open space. | Global  (25 cities) | 500m | Global Human Settlement Layer^85^  OpenStreetMap^236^ |
|  | Urban greenness^214^ | Population-weighted peak and annual mean NDVI.  Cities grouped by urban greenness indicator, HDI and climate region. | Cities were selected based on population size of 500,000 or more.  Calculated population-weighted peak and annual mean NDVI.  Classified cities based on the greenness indicator, climate zone, and level of development.  Repeated analyses for 2010, 2015, and 2020 to facilitate the tracking of urban greenery over time.  Data provided in tabular and graphical format. | Global  (1000 cities) | 1km^2^ | Landsat^257^  Global gridded population^102^  Global Human Settlement Urban Centre^93^  Köppen-Geiger climate classification system^258^  United Nations^259^ |
|  | Green space accessibility^214^ | Urban green space accessibility | For each identified city, constructed accessibility metrics by combining information on population estimates, spatial data on public green areas (utilised for calculating walking distances within two cells in the city) and land cover of green space.  Calculated accessibility indices of minimum distance (to closest public green area), exposure (overall size of available public green space), per-person (m^2^ pre person of public green within walking distance from residential location).  Evaluated the stability of each accessibility index through different parameterisations, including weighting by GINI coefficient; through application of Kendall rank correlation coefficient. | Global  (1000 cities) | 1km^2^ | Global gridded population^102^  Global Human Settlement Urban Centre^93^  OpenStreetMap^247^  World Cover data^82^  Open Source Routing Machine engine^260^ |
|  | Nature based well-being indicator^213^ | Approximates the ‘actual’ subjective quality of nature near people’s homes. | High-amenity nature^a^ identified by combining CORINE data on natural land use with clustered HSM data, on locations of attractive nature.  Spatial cluster analysis conducted on HSM markers identifies natural areas that people have perceived as attractive. It produces a 250m^2^ grid covering the observed country. The density of HSM markers is measured for each individual grid within the larger grid.  Calculates population-weighted mean distance to high-amenity nature. | Netherlands, Germany and Denmark | 250m^2^ | European Environmental Agency (CORINE land cover dataset 2006)^107^  HSM database (Google Maps-based survey tool)^261^ |
|  | Percentage of amenity green space^209^ | Share of land dedicated to green urban areas, sports, and leisure facilities | Calculated the percentage of a city's total land area dedicated to green spaces, sports, and leisure facilities. | Europe  (28 cities) | NUTS3 | Urban Audit^143^ |
|  | Biodiversity of built-up areas^211^ | The percentage of bird species in all areas that were also observed in built-up areas. | Calculated by dividing the number of bird species in built-up areas by the total number of bird species observed across all areas within the city. Built-up areas were delineated using data from the ESA.  To estimate the saturation levels of species-area curves for the number of bird species, utilised research-grade observations of birds between 2016 and 2021.  Calculations were conducted using the observations recorded on built-up land and all observations within city boundaries. | Global | - | ESA WorldCover 2020^115^  iNaturalist database^262^ |
|  | Biodiversity of built-up areas^211^ | The percentage of KBA in built up areas. | Determined the build-up area within a KBA located within a city, and divided this by the total KBA area within the city and multiplied the result by 100. | Global | City-level | ESA WorldCover 2020^115^  Key Biodiversity Areas^263^ |
|  | Proportion of urban terrestrial area^209^ | The percentage of land in a city designated as protected natural areas. | Calculated the percentage of a city's total land area that is designated as protected natural areas. | Europe  (28 cities) | NUTS3 | Urban Audit^143^ |
| **Noise** | Household noise annoyance^209^ | Proportion of population living in households considering that they suffer from noise | Calculated the percentage of the total population who reported being affected by noise. | Europe  (28 cities) | NUTS3 | Urban Audit^143^ |
| **Transport and mobility** | Urban mobility^73^ | Quantifies the hierarchical organisation of urban mobility, considered a proxy for urban inhabitants’ needs being met | Weekly trip flow information of 300 million people aggregated into weighted networks to identify hotspots of activity.  Hotspots enabled analysis of hierarchical organisation in urban mobility and connection to city liveability.  Spatial distribution patterns of hotspots capture differences in city organisation. | Global  (174 cities)  United States  (127 cities) | ~1.27km^2^ and  City-level | United States Census Bureau^264^  Google^265^  Centres for Disease Control and Prevention^266^ |
|  | Local walkability index^205^ | Combines population density, street intersection density, and daily  living destinations in local neighbourhoods. | Calculated population density as the mean of the estimated population density within 1km of local walkable catchments.  Street intersections were calculated as the average of the estimated intersection density within 1km of local walkable catchments.  Daily living score was determined as the sum of binary access indicator scores to supermarkets, convenience stores, and public  transport facilities, serving as a proxy for land use mix.  Walkability index was calculated as the sum of z-scores, both within and between cities, for population density, intersection  density, and daily living score. | Global  (25 cities) | 1km^2^ | Global Human Settlement Layer^93^  Custom boundaries (see Appendix^205^)  OpenStreetMap^250^  General Transit Feed Specification data sources (see Appendix^205^)  World Bank^267^ |
|  | Public transport access^205^ | Percentage of population living within 500m of a frequently serviced public transport stop. | Calculated the percentage of the population living within a 500m radius of any public transport stop. | Global  (25 cities) | 500m | Global Human Settlement Layer^93^  Custom boundaries (see Appendix^205^)  OpenStreetMap^250^  General Transit Feed Specification data sources (see Appendix^205^) |
|  | Length of bicycle network^209^ | Length of dedicated cycle paths and lanes | Calculated the sum of lengths of dedicated bicycle paths. | Europe  (28 cities) | NUTS3^b^ | Urban Audit^143^ |
| **Climate change mitigation** | Greenhouse gas emissions^211^ | The variation in annual greenhouse gas emissions (measured in CO_2_ equivalent [CO_2_ e]) from the city area between 2000 and 2020, expressed as a percentage and broken down by pollutant type and sector. | Sectors include various agricultural activities, power generation, industry, transportation, and waste management.  Using Google Earth Engine, the emissions within city administrative boundaries were calculated, disaggregating the data annually by sector in tonnes/year for 2000 and 2020.  All emissions were converted to CO_2_ equivalent based on 20-year global warming potentials for a standardised measurement. The final indicator presents the percentage change in CO_2_ equivalent emissions from 2000 to 2020. | Global | 11km | Google Earth Engine^255^  CAMS Global  Anthropogenic Emissions^268^ |
|  | Greenhouse gas emissions^209^ | Greenhouse gas emissions from transport (million tonnes) | Calculated the total greenhouse gases measured in equivalent carbon dioxide units, produced by transportation activities in a city over the course of a year. | Europe  (28 cities) | NUTS3 | Urban Audit^143^ |
|  | Climate change impact of trees^211^ | The average annual greenhouse gas net flux from trees (2001–  21) per hectare (ha) of city area (megagrams [Mg] CO_2_e/ha). | Calculated the average annual carbon flux for each area, by assigning a zero value to pixels without carbon flux data.  Mean carbon flux over the area was then calculated and divided by 21 to obtain an annual average for the 21-year period.  This yielded an estimate of the average yearly net carbon flux per hectare for the area of interest.  The entire geographical area, including non-forested regions, was employed for normalisation, with the total area serving as the denominator. Negative numbers indicate net greenhouse gas removals, whereas positive values denote net emissions. | Global | 30m | Google Earth Engine^255^  Net Carbon Flux^269^ |

Abbreviations: OpenStreetMap (OSM); European Space Agency (ESA); Copernicus Atmosphere Monitoring (CAM); National Aeronautics and Space Administration (NASA); Land Surface Temperature (LST); Normalised Difference Vegetation Index (NDVI); Human Development Index (HDI); Hotspotmonitor (HSM); Key Biodiversity Indicator (KBA)

^a^ High amenity defined as one of the following: ecosystem services (ESS), quality of cultural ESS (aesthetics), natural land uses.

^b^ NUTS3: corresponds to small regions or local administrative units that include cities or urban areas^200^.
